# A Review of Micronutrients and the Immune System–Working in Harmony to Reduce the Risk of Infection

**DOI:** 10.3390/nu12010236

**Published:** 2020-01-16

**Authors:** Adrian F. Gombart, Adeline Pierre, Silvia Maggini

**Affiliations:** 1 Linus Pauling Institute, Department of Biochemistry and Biophysics, Oregon State University, 307 Linus Pauling Science Center, Corvallis, OR 97331, USA; adrian.gombart@oregonstate.edu; 2 Bayer Consumer Care AG, 4002 Basel, Switzerland; adeline.pierre@bayer.com

**Keywords:** immune system, infection, micronutrients, minerals, vitamins

## Abstract

Immune support by micronutrients is historically based on vitamin C deficiency and supplementation in scurvy in early times. It has since been established that the complex, integrated immune system needs multiple specific micronutrients, including vitamins A, D, C, E, B6, and B12, folate, zinc, iron, copper, and selenium, which play vital, often synergistic roles at every stage of the immune response. Adequate amounts are essential to ensure the proper function of physical barriers and immune cells; however, daily micronutrient intakes necessary to support immune function may be higher than current recommended dietary allowances. Certain populations have inadequate dietary micronutrient intakes, and situations with increased requirements (e.g., infection, stress, and pollution) further decrease stores within the body. Several micronutrients may be deficient, and even marginal deficiency may impair immunity. Although contradictory data exist, available evidence indicates that supplementation with multiple micronutrients with immune-supporting roles may modulate immune function and reduce the risk of infection. Micronutrients with the strongest evidence for immune support are vitamins C and D and zinc. Better design of human clinical studies addressing dosage and combinations of micronutrients in different populations are required to substantiate the benefits of micronutrient supplementation against infection.

## 1. Introduction

From the moment of birth, our bodies are bombarded by pathogens whose sole purpose is to live and replicate in a warm, moist, nutrient-rich environment. Not all microorganisms are harmful, such as microbiota that have a symbiotic relationship with our gastrointestinal tract [1]. However, many pathogens survive and multiply by using highly specialized mechanisms that enable them to infiltrate the body, find nutritionally compatible niches within to reproduce, then exit and spread to a new host. These processes generate clinical symptoms of disease.

**Figure 1:**
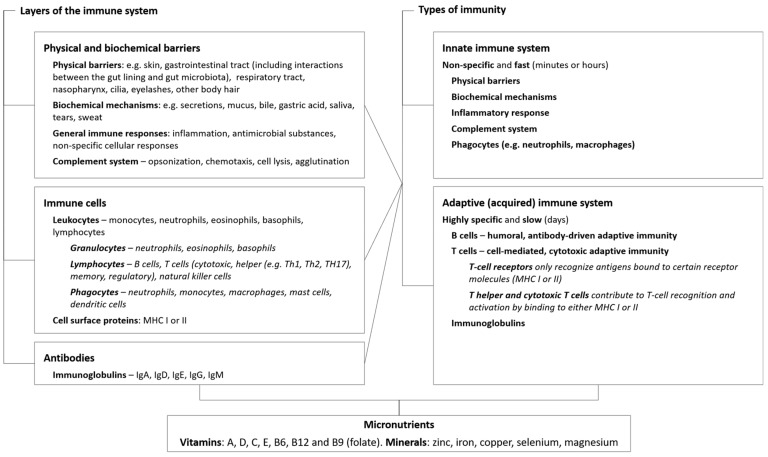
Basic components of the immune system, including key micronutrients that contribute to immune function. The schematic highlights the areas of immunity and the micronutrients that affect these functions that are covered in this review. Abbreviations: Ig, immunoglobulins; MHC, major histocompatibility complex.

To combat pathogenic microorganisms, the elaborate immune defense system comprises physical and biochemical barriers, specialized immune cells, and antibodies that specifically target the pathogen (Figure 1) [2]. The immune system also helps to repair damage caused by noxious insult from external factors, such as environmental pollutants [3] and innate toxins in food (e.g., carotoxins in carrots, persins in avocados, glycoalkaloids in potatoes, and lectins in beans) [4,5]. In brief, the initial onslaught by pathogens or damage by foreign bodies is challenged by the innate immune system. Physical barriers such as the skin, body hair and mucus membranes help to prevent entry into the body. If these are circumvented, biochemical mechanisms quickly identify any “non-self” molecules and destroy and eliminate the threat via myriad immune cells (e.g., leukocytes such as neutrophils, natural killer (NK) cells, and macrophages) and cytokines (involved in cell signaling), then repair any damage. Specific invading agents, such as pathogens and foreign tissues, can activate slower adaptive immune functions that utilize T and B cells. These recognize specific antigens on the invading microorganism and form antibodies against it, which either enable identification for attack by other immune cells or neutralize the pathogen directly.

**Figure 2:**
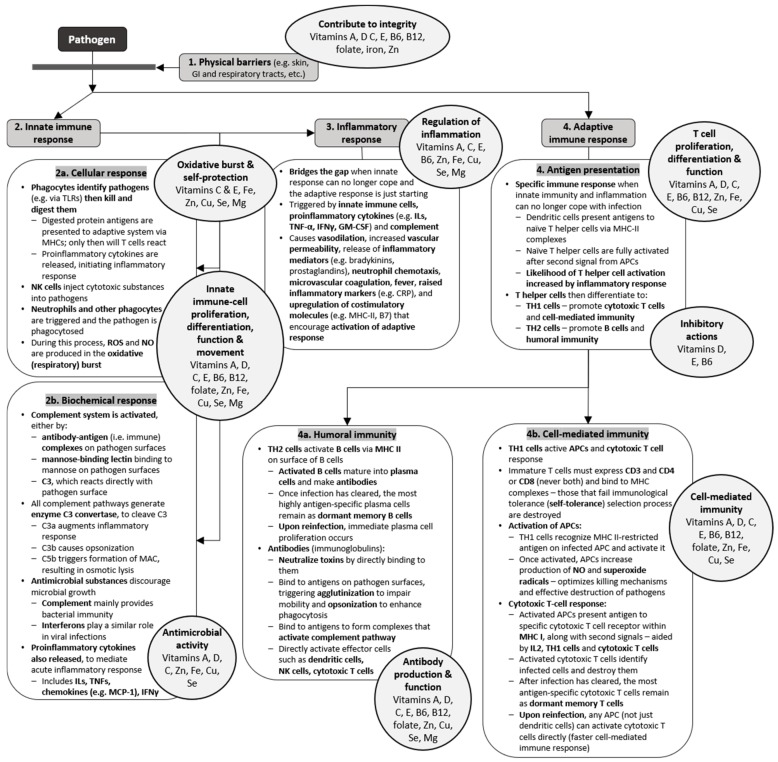
Micronutrients have key roles at every stage of the immune response [2,7,8,9]. This schematic summarizes important components and processes that are involved in different aspects of the innate and adaptive immune responses. The circles highlight those micronutrients that are known to affect these responses. The significant overlap between micronutrients and processes indicates the importance of multiple micronutrients in supporting proper function of the immune system. Abbreviations: APCs, antigen-presenting cells; C3, complement component 3; CRP, C-reactive protein; Cu, copper; Fe, iron; IFNs, interferons; Igs, immunoglobulins; ILs, interleukins; GI, gastrointestinal; GM-CSF, granulocyte-macrophage colony stimulating factor; MAC, membrane attack complex; MCP-1, monocyte chemoattractant protein-1; Mg, magnesium; MHCs, major histocompatibility complexes; NK, natural killer; NO, nitric oxide; ROS, reactive oxygen species; Se, selenium; TLRs, toll-like receptors; TNF, tumor-necrosis factors; Zn, zinc.

Every stage of this immune response is reliant on the presence of certain micronutrients. Historically, the importance of micronutrients in the immune system and on infection was based on vitamin C deficiency and the occurrence of scurvy. In the first recorded controlled clinical trial, published in 1753, James Lind fed different diets to groups of men suffering from scurvy and noted that those who consumed citrus fruit made the most remarkable recovery [6]. Since then, it has been established that several micronutrients are essential to the immune system, and have synergistic roles based on their complementary mode of action (Table 1 and Figure 2). This review provides an overview of the known mechanisms of micronutrients that are fundamental to immune function and outlines the effects of inadequate dietary intakes on the risk of infection. Micronutrient supplementation may be beneficial in such cases to reduce the risk, and the available clinical evidence is presented.

## 2. Micronutrients Are Integral to Immune Function

### 2.1. Physical and Biochemical Barriers

The first line of defense comprises the external and internal surfaces of the body (the skin and all mucus membranes), which form physical and chemical barriers against bacteria, viruses, fungi, parasites, pollen, dust, and toxic chemicals. The structural and functional integrity of the physical barriers require maintenance for optimal function. Micronutrients play vital roles in these processes. For example, iron is essential for differentiation and growth of epithelial tissue [3]. Vitamin A [7] and zinc [3] are important for the structural and function integrity of skin and mucosal cells. The composition of intestinal microbiota (e.g., the balance between commensal and pathogenic microorganisms) is affected by vitamins D [72,73], A, B6, and B12 and folate [10,22,74,75]. Vitamin C is necessary for promotion of collagen synthesis in epithelial tissue [76]. It further enhances keratinocyte differentiation and lipid synthesis as well as fibroblast proliferation and migration [21]. Dietary or exogenous antioxidants such as vitamins C and E, in collaboration with endogenous antioxidant defenses, help to protect cell membranes from damage caused by free radicals generated during normal metabolism, as well as through exposure to toxins and pollutants [7,8,27]. Although reactive oxygen species (ROS) are essential for cell communication [77], for example, at high concentrations they can denature structural and functional cell components such as lipids and proteins [78,79]. Therefore, antioxidants are necessary to reduce increased concentrations to more physiological levels, protecting cells against damage and restoring cell signaling [78,80].

The physical barriers may be covered by hair or hair-like structures and produce fluids rich in chemicals such as sweat, saliva, mucus, bile, and gastric acid; all are designed to trap or filter foreign material and wash or move it (e.g., via coughing or sneezing) out of the body, or contain antimicrobials, acids or enzymes to inhibit the growth of microorganisms or to destroy them. Calcitriol (the active form of vitamin D) stimulates the expression of some antimicrobial peptides in epithelial cells, such as those lining the respiratory tract, protecting the lungs from infection [8,15]. It also stimulates tight junction protein expression, E-cadherin, and connexin 43 in the gastrointestinal tract, which function as structural precursors of gap junctions and provide a communication pathway between the cytosol and extracellular environment of the intestinal barrier [16,17,18]. They also maintain renal epithelial barrier function [19], and enhance corneal epithelial barrier function [20]. The gastrointestinal tract is an important line of defense in which epithelial cells provide a physical and biochemical barrier and work in concert with immune cells and the gut microflora (some of which produce an array of compounds such as bacteriocins) to strengthen the gut barrier, fight off pathogens, and limit their direct contact with the epithelium [81].

### 2.2. Innate Immunity

Invariably, some foreign materials find a way to penetrate these physical and biochemical barriers, but encounter a second line of defense activated by the presence of “foreign” antigen molecules on the invading particles known as pathogen-associated molecular patterns. These defenses include antimicrobial substances in the serum (such as interferons (IFNs) and complement), phagocytes, and NK cells, all of which have a role in the inflammatory process.

#### 2.2.1. Antimicrobial Substances

There are several antimicrobial substances that discourage microbial growth. Interferons defend the body by preventing viral replication. Selenium supplementation of adults (50 or 100 µg per day for 15 weeks) increased the production of IFNγ [82], while vitamin A downregulates it; vitamin C, zinc and iron may also play a role in its production [3,21]. Complement proteins mark pathogens and enhance opsonization by phagocytes. They also attract other immune cells from the blood, disrupt the cell membranes of bacteria, and fight viruses by destroying their envelopes or cells infected by viruses. The serum levels of complement proteins are increased by vitamin C [7], and zinc inhibits complement activity [83].

Iron-binding proteins (e.g., transferrin, lactoferrin, ferritin, and hemoglobin) sequester nutritional iron and inhibit the growth of certain bacteria. Defensins and cathelicidins are antimicrobial peptides synthesized in neutrophils, monocytes, and NK cells. They can kill a range of microbes, particularly bacteria, and attract antigen-presenting dendritic cells (DCs) and mast cells [84]. Calcitriol, the active form of vitamin D, regulates the expression of these potent antimicrobial peptides [39,40,41], while copper has intrinsic antimicrobial properties that destroy a wide range of microorganisms [7].

#### 2.2.2. Natural Killer Cells and Phagocytes

If pathogens bypass the antimicrobial defenses, NK cells are activated to target and attack any host cells that display abnormal or unusual plasma membrane proteins, and kill the cells using cytotoxins. Vitamin A helps regulate the number and function of NK cells, while vitamins B6, B12, C, and E, folate, and zinc maintain or enhance their cytotoxic activity [2,8,27].

When NK cells kill infected cells, the microbes inside are released and destroyed through phagocytosis by neutrophils and macrophages, which migrate to infected areas. Neutrophils predominate in the early stages of infection but die off rapidly. Monocytes follow the neutrophils and, once in the tissue, enlarge and develop into actively-phagocytic macrophages. Tissue-resident macrophages perform tissue-specific functions ranging from immune surveillance, response to infection, resolution of inflammation, and clearance of cellular debris [85]. Macrophages are known to contain significant amounts of iron [86], while monocytes and macrophages (and DCs and thymus) contain vitamin D receptors [8]. Activated macrophages can synthesize calcitriol from circulating calcidiol, and calcitriol increases the proliferation and differentiation of monocytes to macrophages [8,27,87]. Calcitriol also promotes the movement and phagocytic ability of macrophages [27], and increases their oxidative burst potential [24,25,26]. Vitamin C stimulates the production and function of leukocytes, particularly the movement of neutrophils and monocytes, and is readily mobilized during infection [24]. In a recent study, neutrophils isolated from healthy individuals were preloaded with dehydroascorbic acid to increase intracellular vitamin C levels [28]. These elevated levels did not affect superoxide production or chemotaxis but did attenuate neutrophil extracellular trap (NET) formation, suggesting that higher oral intakes of vitamin C may reduce tissue damage associated with NET formation. Magnesium modulates the activation of peripheral blood neutrophils and eosinophils from eosinophilic patients, with high concentrations of magnesium reducing superoxide anion production [59].

Phagocytosis by immune cells involves several highly-coordinated steps. Initially, phagocytes migrate to the infected area and adhere to the microbe or foreign material, enhanced by complement proteins. The phagocyte engulfs the microbe and digests it, producing ROS in the oxidative or respiratory burst. Finally, a combination of digestive enzymes, antimicrobial peptides and proteins, and oxidants kill the microbe within the phagosome. Vitamin A contributes to the phagocytic and oxidative burst activity of macrophages, while calcitriol increases phagocytosis, superoxide synthesis, and bacterial killing [8]. The antioxidant activities of vitamins C and E are essential to protect against free radical damage during the oxidative burst [2], and high levels of vitamin C in neutrophils are necessary to counteract the high levels of oxidative stress [8]. Vitamin C also regenerates antioxidants including vitamin E and glutathione to their active state [2], and has roles in apoptosis and clearance of spent neutrophils by macrophages from the sites of infection [21]. Declining T-cell function is a hallmark of immunosenescence and likely due to production of T cell-suppressing prostaglandin E2 from macrophages [88]. In animal models, vitamin E intervention reverses these changes by inhibiting PGE(2) production by macrophages, thus indirectly protecting T-cell function [27,29]. Selenium is essential for the function of selenoproteins, which act as redox regulators and cellular antioxidants and are thus important for the function of leukocytes and NK cells [35]. It also helps (via glutathione peroxidase) to protect against oxidative stress by removing excess and potentially damaging free radicals [8]. Zinc enhances the phagocytic activity of peritoneal macrophages for *Escherichia coli* and *Staphylococcus aureus* [31] and zinc deficiency decreased phagocytic capacity of monocytes in children suffering from *E. coli*-induced diarrhea, but supplementation improved it [27,32]. The role of zinc in modulating oxidative burst activity is unclear [31]. Zinc has antioxidant effects that protect against ROS and reactive nitrogen species [49], and it influences the activity of antioxidant proteins [8]. The generation of ROS by neutrophils to kill pathogens requires iron [2]. Activation of the NADPH oxidase complex generates superoxide anion (O_2_^−^) that undergoes dismutation to hydrogen peroxide (H_2_O_2_). Through the Fenton reaction, H_2_O_2_ oxidizes ferrous iron to generate highly reactive OH^−^ [89]. Through a Fenton-like reaction, copper can also catalyze the formation of ROS and is thus used to kill pathogens [8,90], while it also acts as a free-radical scavenger [58]. Magnesium helps to protect DNA against oxidative damage and is involved in the regulation of apoptosis [37].

Phagocytosis can be mediated by cytokines such as tumor necrosis factor (TNF), mainly produced by macrophages, which stimulates accumulation of neutrophils and macrophages at sites of inflammation and stimulates them to kill microbes. The production of TNF-α is increased by calcitriol [8,91].

#### 2.2.3. Inflammatory Response

Tissue can be damaged by pathogens or other factors such as toxins, pollutants, trauma, and extreme temperatures. This elicits an inflammatory response that removes any foreign material at the site of injury, significantly reduces the spread of pathogens to other tissues, and prepares the site for tissue repair. The fever that can accompany inflammation intensifies the effects of IFNs, inhibits some microbial growth, and speeds up the body reactions that aid repair [92]. Following injury, vasoconstriction of the capillaries occurs briefly followed by vasodilation, resulting from histamine release from mast cells, basophils, and platelets to increase blood flow and vascular permeability. These physiological responses can dilute bacterial products and toxins at the site of injury or infection and promote an influx of phagocytes that amplify the inflammatory response when activated by the presence of PAMPs or opsins on pathogens. The activated phagocytes release pro- and anti-inflammatory cytokines/chemokines, bradykinin, prostaglandins, leukotrienes, and complement, which keeps capillaries dilated, floods the tissues with fluids, and increases the numbers of neutrophils to fight pathogens. Subsequently, macrophages are recruited to clean up the dead cells and debris and healing occurs [92].

Vitamin A helps to regulate the production of IL-2 and the proinflammatory TNF-α, which activates the microbial action of macrophages [8]. Administration of vitamin D reduces the expression of pro-inflammatory cytokines and increases the expression of anti-inflammatory cytokines by macrophages via upregulation of MAPK phosphatase-1 and suppression of p38 activation [24,46,47,48]. Vitamin E decreases the production of prostaglandin E2 (which has immunosuppressive activity) [8], and vitamin C modulates cytokine production and decreases histamine levels [21]. In 2229 adults enrolled in the Framingham Offspring study, those with the lowest levels of pyridoxal 5′-phosphate (PLP), the active form of vitamin B6, had the highest levels of chronic inflammation, whereas those with highest levels of PLP had the lowest inflammation scores [51]. PLP is a cofactor in more than 150 enzymatic reactions and may help regulate inflammation by acting in pathways that produce metabolites with immunomodulatory effects [52]. In vitro and in vivo studies show that an iron-rich status promotes an M2-like macrophage phenotype (which is associated with wound healing and tissue repair) and negatively regulates an M1 pro-inflammatory response (such as production of ROS) through reduced NF-kB p65 nuclear translocation [33]. Zinc is an anti-inflammatory agent [8], while copper is important for the production and response of IL-2 to adaptive immune cells and accumulates at the sites of inflammation [2].

### 2.3. Adaptive Immunity

Adaptive immunity is a much slower process that defends the body against specific invading agents, again provoked by antigens. T and B cells (lymphocytes) develop in red bone marrow and either mature there (B cells) or in the thymus (T cells). There are three kinds of mature T cells. Cytotoxic CD8^+^ T cells kill their target cells after recognition of peptide antigens complexed with major histocompatibility complex (MHC) molecules on the target-cell membrane. CD4^+^ T helper (Th) cells aid B and other T cells to fulfil their functions. Regulatory T cells (Tregs) are a specialized subpopulation of T cells that are important for the induction and maintenance of peripheral tolerance; therefore, they are key in preventing excessive immune responses and autoimmunity [93]. Depending on the cytokines that they secrete and the immune responses they generate, Th cells are further differentiated into Th1 and Th2 cells. Th1 cells primarily produce IFNγ and IL-2 and tend to initiate responses against intracellular bacteria and viruses; Th2 cells secrete several other ILs (IL-4, IL-5, IL-10, and IL-13), and trigger immune responses against extracellular microorganisms. The development and differentiation of Th1 and Th2 cells is dependent on vitamin A, which supports the Th2 anti-inflammatory response by suppressing IL-12, TNF-α, and IFNγ production of Th1 lymphocytes [3,8]. In contrast, vitamin E is known to suppress the Th2 response [3,35]. Vitamin E, as well as vitamin B6, folate, and zinc, maintain the Th1-mediated immune response, while vitamin D inhibits Th1-cell activity [8,94,95].

Before the first exposure to a given antigen, only a few lymphocytes can recognize it; these lymphocytes undergo clonal selection (i.e., proliferation and differentiation) to form a population of identical, specialized cells that can recognize the same specific antigen as the original lymphocyte. Lymphocyte differentiation and proliferation is modulated by vitamins C, E, and B6, while vitamin B6 also has a role in their maturation [2,7]. Vitamin D is an immunosuppressive hormone [62]; calcitriol inhibits the proliferation and differentiation of T and B cells [7,27], but production of calcitriol by DCs “programs” T-cell homing to the epidermis, which is essential for long-term immune surveillance and maintenance of the barrier integrity [61,96]. In contrast, vitamins C and B12 facilitate the production of T cells, particularly cytotoxic T cells [24,35]. Zinc is also essential for the development, differentiation, and activation of T lymphocytes [8,49], while iron, copper, and selenium are important in their differentiation and proliferation [2,7,35]. The acquisition of mucosal-homing properties by T and B cells is mediated by vitamin A [8].

The adaptive immune response is mediated by T and B cells: effector cells (active Th cells, active cytotoxic T cells, and plasma cells), which eventually die after the immune response is completed, and memory cells (memory Th cells, memory cytotoxic T cells, and memory B cells), which have long lifespans often lasting for decades. The adaptive immune response involves either antibody or cell-mediated responses to clear pathogens. In antibody responses, activated B cells secrete antibodies that circulate in the blood and fluids to mark pathogens for destruction by phagocytes. In cell-mediated responses, activated T cells kill host cells that present foreign antigens on their cell surface or they stimulate other immune cells to kill pathogens. If the antigen reappears in the body, both cell-mediated and antibody-mediated immune responses are much quicker and intense; within hours, memory T cells are able to proliferate and differentiate into Th cells or cytotoxic T cells, and B cells into plasma cells. Vitamin D inhibits the effector functions of T helper cells and cytotoxic T cells, but promotes the development of Tregs that dampen immune-mediated inflammation [27,62,63].

#### 2.3.1. Antigen Recognition

The adaptive immune response begins with recognition of an antigen as self or non-self. All nucleated cells and platelets, but not red blood cells, in the body possess MHC class I (MHC-I) molecules at the cell surface that present self-antigens, while class II MHC (MHC-II) antigens appear on the surface of antigen-presenting cells (APCs, including DCs, macrophages, and B cells) that primarily internalize exogenous antigens. The APCs fragment the antigens into peptides that associate with MHC-II molecules and insert into the plasma membrane of the cell for antigen presentation. Calcitriol is known to promote antigen processing [8], while vitamin D has a role in the down-regulation of MHC-II [35]. Calcitriol has an inhibitory effect on the differentiation and maturation of DCs, and helps program DCs for tolerance [27,64,65,66]. Magnesium plays a key role in antigen binding to macrophages [38].

Each unique T-cell receptor (TCR) is able to recognize a specific antigen–MHC complex. Antigen recognition by a TCR is the first signal in activation of a T cell. T cells ignore complexes that come from a self-antigen but trigger an immune response if the antigen fragment comes from a foreign protein–a process accompanied by a second signal, such as the presence of IL-2. The production of IL-2 is enhanced by vitamin E [3], and zinc is important in maintaining immune tolerance as it induces the development of Treg cells [97,98] and dampens the development pro-inflammatory Th17 and Th9 cells [27,54,55].

#### 2.3.2. Cell-Mediated Immunity

During cell-mediated immunity, activated T cells kill host cells that present foreign antigens on their cell surface or they stimulate other immune cells to kill pathogens. Initially, resting Th cells recognize exogenous antigen–MHC-II complexes at the surface of an APC, interact with the APC (with the aid of the CD4 protein), receive the second signal (e.g., IL-2), and become activated. Vitamin E helps to form effective immune synapses between the APC and Th cells [27]. The activated Th cell undergoes clonal selection to form a population of active and memory Th cells. Active Th cells then begin to secrete a variety of cytokines such as ILs (which can act as a costimulator for B cells, cause plasma cells to secrete antibodies, or activate NK cells). IL-2 is required for virtually all immune responses, and is the prime trigger of T cell proliferation. The production and activity of cytokines is regulated by iron [9]. Vitamin E enhances IL-2 production [8], whereas vitamin D inhibits the production of IL-2 [99,100,101] and IFNγ [27,42,43,44,45]. Copper has a role in cellular immunity (including T-cell proliferation and NK activity [36], and copper deficiency reduces IL-2 expression in human T-cells [7,35]). Zinc influences the generation of cytokines such as IL-2, IL-6, and TNF-α [56,57]. T-cell activity is influenced by vitamins E and B6 and by zinc homeostasis [8], and by a possible synergistic effect between vitamin A and zinc [102].

Cytotoxic (CD8^+^) T cells recognize endogenous antigen–MHC-I complexes on the surface of infected body cells and are activated by IL-2, IFN, or other cytokines produced by active Th cells. Once activated, the cytotoxic T cells undergo clonal selection and expansion into active and memory cytotoxic T cells. The proliferation of cytotoxic T cells is induced by zinc [2], while vitamin B12 may act as an immunomodulator and enhance the number of cytotoxic T cells [3,8]. The active cytotoxic T cells attack host cells displaying that antigen, while memory cytotoxic T cells quickly proliferate and differentiate into additional active and memory cytotoxic T cells.

#### 2.3.3. Antibody-Mediated Immunity

Antibody-mediated (humoral) immunity mainly works against extracellular pathogens in extracellular body fluids, such as blood and lymph. During this process, B cells break down the antigen, combine it with MHC-II self-antigens, and move the resulting complex into the B-cell plasma membrane. Th cells recognize the antigen-MHC-II complex and produce IL-2 and other cytokines to activate the B cells. Once activated, the B cell undergoes clonal selection and expansion into plasma cells and memory B cells. Plasma cells synthesize and secrete antibodies, which bind to a specific antigen, while memory B cells do not secrete antibodies but instead quickly proliferate and differentiate into more plasma cells and memory B cells if the antigen reappears in the future. Antibodies (and cytokines) are synthesized from amino acids; thus, like all proteins, they require vitamins B6 and B12 and folate during their endogenous synthesis and metabolism [7,8,35]. In patients with B12 deficiency, decreased CD8^+^ cells levels were observed, as was a high CD4/CD8 ratio and suppressed NK cell activity [103]. B12 is necessary for cell replication and cell division and this may explain the effect it has on rapidly proliferating B cells. Vitamin C increases serum levels of antibodies [7,21], and both copper and selenium have roles in antibody production [9,35]. Magnesium also acts as a cofactor for the synthesis of antibodies [38]. Calcitriol has an inhibitory effect, and suppresses IL-2 driven B-cell antibody production [7,8].

Antibodies neutralize the antigen, immobilize bacteria, and agglutinate pathogens, thus enhancing ingestion of microbes by phagocytes; antibodies also activate complement, which increases phagocytosis by attracting phagocytes to the site of infection. Generation of an appropriate antibody response to antigen requires adequate amounts of folate [35] and vitamin A (which influences the proper functioning of B cells) [7]. For example, B cells activated with retinoic acid (a metabolite of vitamin A) produced by gut-associated, lymphoid tissue DC cells express high levels of gut-homing receptor, a factor that may contribute to the balance between immunity and tolerance in the intestine [104]. Furthermore, vitamin A is necessary for B cell-mediated IgA antibody responses to bacterial antigens [104]. Zinc is involved in antibody production, particularly IgG [69,70]. Microarray analysis of T-lymphocyte population changes in moderate zinc deficiency showed changes in expression of 1200 genes related to the proliferation, survival, and response of T-cells [105].

## 3. Impact of Micronutrient Status on the Immune Response and Risk of Infection

The body requires optimal levels of micronutrients for effective immune function, with different requirements throughout every stage of life [2]. It is well established that overt (clinical) micronutrient deficiencies adversely affect the immune system and predispose individuals to infections [58,106,107,108] (Table 2). For example, micronutrient deficiency is known to increase the risk of morbidity and mortality associated with measles, pneumonia, and diarrheal disease [58,109,110,111]—all common infections encountered worldwide and among the leading causes of death [112]. Even in industrialized countries, multiple micronutrient deficiencies are widespread and may exacerbate the risk of infection [113,114]. The severity of any adverse health effects largely depend on the extent and duration of micronutrient deficiency [24].

**Figure 3:**
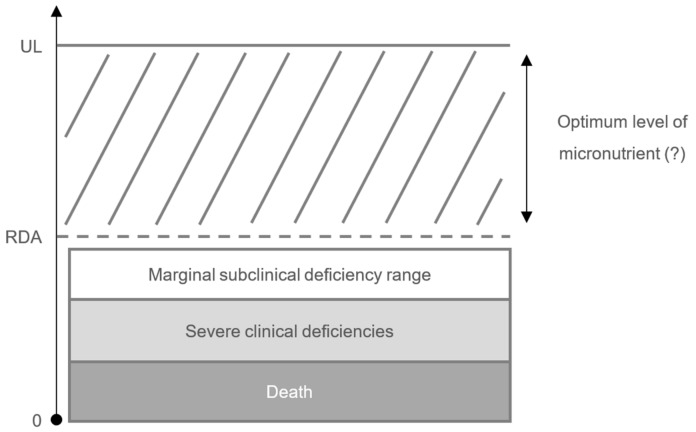
For optimal immune protection and resistance to infection, daily intakes may need to be much higher than the RDAs [24,27]. Abbreviations: RDA, recommended dietary allowance [143]; UL = tolerable upper intake level, the maximum daily intake unlikely to cause adverse health effects.

There is much less information on health effects when micronutrient status is suboptimal (rather than clinically deficient), including what levels can be defined as “suboptimal” (particularly in different populations in whom “optimal” levels may vary, i.e., the levels that would provide optimized function or protection against health risk) [24], and how to define the levels of a micronutrient that are optimal in terms of immune function. Most people are aware of the recommended dietary allowance (RDA) for all nutrients, which is the average daily level of intake that is necessary to avoid clinical or subclinical deficiency in the majority (97–98%) of a healthy general population (Table 3) [143]. As useful as these reference values are to those planning and assessing nutrient intakes, the scarcity of data means that it is not currently possible to give an indication of the levels required to optimize immune protection and resistance to infection [144]. It may be that these values are much higher than the RDAs [24,27] (Figure 3). For example, in the case of vitamin C the RDA ranges globally between 40 and 110 mg [145]. However, prophylaxis of infection requires dietary vitamin C intakes of 100–200 mg/day (i.e., higher than the RDA) to provide adequate, if not saturating plasma levels and thus optimize cell and tissue levels [21]. Treatment of established infections requires even higher doses (possibly around 6 g/day [146]) to compensate for the increased inflammatory response and metabolic demand [21]. In older people, studies use high doses of vitamin E, suggesting that intake above the currently recommended level may help to restore T-cell function [29,124,147] and vaccine efficacy (which declines with aging) [148]. One study has shown that a daily supplementation with 200 IU of synthetic α-tocopherol for one year significantly lowered the risk of contracting upper respiratory tract infections [149]. With regard to vitamin B6, supplementation in young women in doses up to 2.1 mg/day (i.e., higher than the RDA of 1.3 mg/day [143]) for one week increased lymphocyte proliferation in a dose-dependent manner [150]. Furthermore, studies investigating the role of selenium again viral infection suggest that supplementation with selenium up to 200 μg/day (i.e., higher than the RDA of 55 μg/day in adults [143]) can be used as safe adjuvant therapy in viral infections (e.g., HIV, type A influenza virus), as well as in coinfections by HIV and *M. tuberculosis* to help viral shedding, to support chemotherapy and/or to improve fitness and quality of life of the patients [151]. A significant body of work suggests that the RDA for vitamin D is unlikely to raise serum levels needed for adequate function of the immune system [152,153].

However, gaps already exist between micronutrient intakes and the minimal, possibly conservative requirements set by the RDA. For example, deficiencies in vitamin A, iron, and iodine are common, particularly in developing countries [154], and are among the leading contributors to the global burden of disease [112]. Vitamin D and calcium deficiencies are also prevalent, which increases the risk of rickets [155]. Potassium intakes are also recognized to be inadequate in most countries, which may increase the risk of hypertension, and cardiovascular disease [156]. A significant proportion of the general population in industrialized nations has inadequate intakes of certain micronutrients [114,157,158], and multiple micronutrient deficiencies frequently occur simultaneously in children and adults worldwide [159,160]. The most recent data from the European Nutrition and Health Report and the United States Department of Agriculture indicates that, depending on the micronutrient, roughly 25–50% of people have an adequate intake or even a surfeit of many micronutrients. Nevertheless, roughly 25–75% of people have a dietary intake that is less than the RDA, depending on the micronutrient [114,158]. In Europe, reported intakes were inadequate for vitamins D and E, folate, and selenium throughout all age groups. Intakes were also inadequate for: vitamin A, zinc, and magnesium in children >10 years and adults; vitamin C in boys >10 years and adults; iron in children and adults, but not older adults; vitamin B12 in adults; and vitamin B6 in older adults [114] (Table 3). In the US, dietary intake for all micronutrients appears to be less than the estimated average requirements or the adequate intake, but particularly for vitamins A, D, and E, calcium, magnesium, zinc, and potassium in all adults; vitamin C especially in adult smokers; vitamin B6 in older people; folate in females; and copper in females [158]. It is also important to consider the dietary source of micronutrients in the assessment of micronutrient inadequacies, as it may strongly impact the bioavailability of micronutrients. For example, it is well known that the bioavailability of trace elements such as iron, zinc, or magnesium in a plant-based diet is low, mainly because of the presence of components that inhibit mineral bioavailability [161,162,163,164,165,166].

Many factors can affect micronutrient status. Lack of nutritious food or certain food groups because of limited availability, income, or lifestyle choices (e.g., vegetarianism or veganism) has an impact. A stressful lifestyle, often accompanied by lack of sleep and reduced physical activity, can increase oxidative stress and thus increase the need for antioxidants such as vitamins C and E, as well as magnesium to help repair DNA [37]. Certain health conditions, such as diabetes and obesity, also have an adverse effect on micronutrient status [167,168], as do genetic factors/polymorphisms (e.g., on fat soluble vitamins such as cholecalciferol) [169,170]. Seasonal changes can decrease micronutrient levels, with lower serum concentrations of vitamin D, for example, in the dark months of winter or in northern climates, or conversely in hot countries where vitamin D absorption is blocked by sunscreen or protective clothing [24]. Multiple micronutrients (e.g., magnesium, zinc, and iron) may be lost during sweating in hot countries or during exercise; these cannot be synthesized in the body and need to be replaced via the diet [24]. In fact, increased energy expenditure utilizes the body’s store of micronutrients to produce more energy, resulting in low levels of B vitamins, vitamin C, calcium, iron, zinc, and magnesium in active individuals [24]. Air pollution may also reduce the body’s concentrations of certain micronutrients, such as vitamin D if the pollution reduces exposure to the sun and thus cutaneous production [171,172], or antioxidants such as vitamins C and E, which may be necessary to combat oxidative stress caused by pollution [3]. 

The body may also lose micronutrients when exposed to pathogens, which causes the immune system to become increasingly active [106]. The loss is exacerbated during an active infection (including vitamins A, C, and E, calcium, zinc, and iron), and plasma levels only return to normal once symptoms improve [24]. An adequate micronutrient intake is essential to aid recovery from infection, made more difficult by the fact that food intake may decrease during illness, and that antibiotic use can also deplete certain micronutrients [24]. For example, levels of vitamin C in plasma rapidly fall to half their original concentration during an infection [146], to levels indicative of a suboptimal status with a risk of deficiency (i.e., ≤50 μmol/L [173]) [174]. However, the high intake of vitamin C required to counteract the fall in concentration after infection (gram doses [146]), or even simply to help reduce the risk of infection (100–200 mg/day) [21], may be difficult to achieve when the data show that people already often fail to reach the current RDA for vitamin C (25–90 mg/day, depending on age [143]), and that an inadequate vitamin C intake is already more widespread than many people realize [114,158].

## 4. Effects of Supplementation on the Risk of Infection

There is clearly a rationale to supplement dietary intake with micronutrients. As discussed, vitamins and minerals have varied roles throughout every stage of the immune system and the immune response is likely to be impaired when micronutrient levels are insufficient. Data suggest that many people have an inadequate daily intake of micronutrients, even when nutritious food is more easily available. Supplementing the diet with deficient micronutrients has been shown to improve various specific immune functions (Table 2), while supplementation with multiple micronutrients (MMN) may also have significant benefits on immune cells and responses [175,176,177,178]. It should be noted that the source of the micronutrient supplement should be considered (i.e., dietary versus supplements), especially for minerals; organic forms of selenium are more bioavailable than inorganic compounds [179], while the sulfate, gluconate, and fumarate salts of iron have good availability [154], unlike its oxide forms [180]. The bioavailability could potentially have an effect on the efficacy of the supplement.

The question remains whether there any clinical benefits to supplementation with vitamins and/or minerals, either singly or in the form of an MMN supplement. For example, does micronutrient supplementation have any effect on reducing the risk or in the management of acute infections? Benefits have been reported in individual studies, which suggest that micronutrients may have the potential to restore resistance to certain types of infections (for examples, see [149,181,182,183,184,185,186,187,188]).

However, a better representation of any clinical effects is achieved when the results from all relevant studies are pooled and analyzed, as in the case of systematic reviews and meta-analyses that generally comprise the most robust studies available. Many such analyses are outlined in Table S1, with varying and sometimes contradictory results for both risk reduction and management, as described below. These variations occur because of the inconsistent study designs, different populations used (for example, the effects of supplementation may be greater in undernourished and older populations [189]), and differences in the type, dosage and source of micronutrients studied. It should be noted that different definitions were used within the analyses for “low”, “moderate”, and “high” quality studies.

### 4.1. Micronutrients in Reducing the Risk of Acute Infections

**Vitamin A.** There is low-to-moderate evidence that vitamin A supplementation (50,000–200,000 IU every 4–6 months) in children can reduce the incidence of diarrhea and measles [190]. However, other analyses in children did not find that vitamin A significantly reduced the incidence of pneumonia or lower respiratory tract infections (RTI) [191,192].

**Vitamin D.** Five meta-analyses of mostly high-quality studies demonstrated that vitamin D (300–3653 IU/day) in adults and children can reduce the risk of RTI [120,193,194,195,196]. Better results were achieved in those with a low vitamin D status at the start of the trial [120,193], with a lower odds ratio for risk reduction with low (0.30) versus high (0.75) vitamin D status [193]. Low-to-moderate quality evidence supports the potential benefits of vitamin D supplementation in reducing the risk of upper RTI, tuberculosis, and influenza [197] in adults and children, although other analyses found no such effect against RTI [198,199], pneumonia [120,199,200], tuberculosis [120]), or diarrhea [200].

**Vitamin C.** The effects of vitamin C in reducing the risk of common cold have long been debated. One analysis of mostly high-quality studies determined that there was no reduction in incidence in the general population, but that vitamin C supplementation (≥0.2 g/day) in those who regularly undergo severe physical exercise reduced the incidence of common cold by more than half [201]. Low-quality evidence further supports a reduced risk of upper RTI in athletes after vitamin C supplementation (0.3–2.0 g/day), with no additional benefits from the addition of vitamin E or zinc [202]. A significant reduction in the risk of pneumonia has been reported after vitamin C supplementation in adults and children, particularly when dietary intake was low (low-to-moderate quality studies) [203]. Finally, there is low-to-moderate evidence to suggest that vitamin C (100 mg/day) supplementation during pregnancy may reduce the risk of urinary tract infections [204].

**Zinc.** Mostly high-quality evidence indicates that supplementation with zinc (5–50 mg/day) can reduce the incidence of otitis media in younger or undernourished children [205]. A reduction in the incidence of lower RTI after zinc supplementation (20–140 mg/week) in children is supported by low-to-moderate evidence, but this outcome depends on the criteria used to define lower RTI; a greater reduction was observed using specific clinical criteria, compared with those based on caregiver reports or “non-severe pneumonia” from the World Health Organization [206]. An analysis of mostly high-quality studies has demonstrated that the risk of RTI or pneumonia and diarrhea or dysentery may be reduced in children after zinc administration [207]. However, analysis of low-to-moderate quality studies found no protective effect of zinc (5 to ≥20 mg/day) against the risk of RTI or malaria in children, although there was a reduction in mortality associated with RTI, diarrhea, and malaria; the addition of iron to zinc conferred no additional benefits [208,209].

**Iron.** Moderate and high evidence indicates that iron supplementation in children reduces the risk of RTI, but not the overall risk of infection or other illnesses such as diarrhea or malarial parasitemia [210].

**MMN.** In children, low-to-moderate quality studies demonstrate that MMN supplementation may reduce the risk of infection and reinfection from helminths [211]. MMN supplementation resulted in significantly fewer episodes of infection in younger adults [189] (low-to-moderate evidence). In older adults, MMN supplementation reduced the mean number of days spent with infection [212], but did not appear to have any beneficial effects on the overall number of episodes experienced [189] (all low-to-moderate evidence). It was stated that supplementation may be more beneficial in older adults if they were undernourished and supplemented for more than six months [189].

### 4.2. Micronutrients in the Management of Acute Infections

**Vitamin A.** Supplementation with vitamin A in children after non-measles pneumonia can reduce the recurrence of bronchopneumonia and time to remission (low-to-moderate quality evidence) [213]. Low-to-moderate evidence also suggests there is a significant reduction in deaths from diarrheal and respiratory diseases associated with measles after administration of vitamin A in children [214]. However, vitamin A after pneumonia in children was unable to significantly reduce mortality [213], the duration of illness [191], or the duration of hospital stay [191,213].

**Vitamin D.** Low-to-moderate evidence suggests there may be potential benefits of supplementing vitamin D in adults and children with tuberculosis, influenza, or upper RTI [197]. However, inconclusive results were observed after supplementation with vitamin D as an adjunct to antibiotics in children with pneumonia; there was a significant reduction in the duration of hospitalization, but only slight benefits on the resolution of acute illness and mortality rate, and no beneficial effects on fever [215].

**Vitamin C.** Mostly high-quality evidence demonstrates that vitamin C supplementation (≥0.2 g/day, or therapeutic doses of 4–8 g/day) in adults and children with a common cold can significantly reduce its duration [201,216] and severity [201], shorten the time of confinement indoors [216], and relieve cold symptoms including chest pain, fever, and chills [216]. The greatest benefits may be seen in children, although no therapeutic trials have looked at the effects of vitamin C in treating common cold in children; there were no consistent benefits in specific therapeutic trials in adults [201]. In older people with pneumonia, vitamin C can significantly reduce the severity of disease and the risk of death, especially if plasma levels are low initially (low-to-moderate evidence) [203]. The duration of pneumonia may also be reduced after vitamin C supplementation in adults, an effect that is dose-dependent [203]. No benefits have been observed in hospital-acquired pneumonia in adults after supplementation with vitamin C [203], or in the eradication rate of *Helicobacter pylori* with either vitamin C with vitamin E or vitamin C alone in adults [217,218].

**Zinc.** The duration of the common cold may be reduced in adults and children after administration of zinc >75 mg/day, but not at lower doses; the type of zinc salt used can also have an effect, with greater benefits with zinc acetate compared with other zinc salts (mostly high-quality studies) [219]. However, zinc (10–20 mg/day) had no significant effect on pneumonia in children, failing to reduce the time to recovery from severe pneumonia [220,221], duration of hospital stay [220,221,222], time to clinical recovery [222], or time to recovery from the effects of severe pneumonia including tachypnoea and chest indrawing (all low-to-moderate evidence) [221,222].

**MMN.** Supplementation with MMN up to ten times the dietary reference intake has not been shown to have a beneficial effect in adults and children with tuberculosis, with insufficient evidence to indicate whether MMN improves “cure”, “treatment completion”, or the “proportion of people who remained sputum positive during the first 8 weeks” (low-to-moderate quality studies) [223].

## 5. Future Directions

Both single supplementation studies and preliminary data on MMN indicate some efficacy in treating and reducing the risk of infections, while others show no overall benefits (Table S1). However, many of the individual studies that comprise these analyses are subject to certain limitations, whether they report positive or negative findings. Many of the studies are of low or moderate quality, and are often small trials with poor methodology and inconsistent reporting of efficacy and safety outcomes. More importantly, the micronutrient status of participants before supplementation is not always recorded. Several are conducted in low- to middle-income countries, making it more difficult to generalize any results to developed countries.

Furthermore, it has been stated that “we can expect only limited success in controlling the effects of micronutrient deficiencies by tackling one micronutrient at a time”, arguing for increased multiple micronutrient intake either via the diet, fortification (such as with vitamin D, which has greatly reduced the risk of rickets in children, with no safety issues [224]), or supplementation [225]. This is in line with the fact that micronutrient deficiencies do not occur in isolation, and that multiple micronutrients are required to support immune function. It has also been suggested that “increased intake above currently recommended levels may help optimize immune function including improving defense function and thus resistance to infection, while maintaining tolerance” [27]. On the other hand, it has been argued that supplementation with micronutrients is useless and a waste of money [226]. A number of researchers from several research institutions subsequently argued that this is not the case, that the claim is wrong and misinforms the public ([226], correspondence).

Thus, further research in standardized, better-characterized clinical trials is urgently required to further investigate the possible effects of supplementation on the risk of infection and its management in different types of populations, considering their micronutrient status. For example, micronutrients have multiple roles throughout the immune response, yet dietary intakes are often inadequate. Is supplementation beneficial in people with marginal, possible multiple micronutrient deficiencies (as opposed to those with more severe deficiencies)? What is the daily intake of micronutrient(s) required to optimize immune function and confer better protection against infection (as has already been determined for vitamins C and D)? Will supplementing with doses higher than the RDA provide greater benefits? Will an MMN supplement containing micronutrients with demonstrable immune-supporting effects have clinical benefits, and can these be replicated in different populations? What is the role of immune-supportive substances such as phytochemicals? The development of biomarker approaches to further capture the broad-spectrum and complexity of the immune response such as metabolomics, as well as non-invasive assessment of immune function and nutrient status, would help to better understand the role of micronutrients on the immune function.

Considering that many people have suboptimal levels of not just one but several micronutrients [113,114], and that immune defenses require a number of micronutrients for optimal functioning, the role of MMN supplementation in treating and reducing the risk of infections needs to be elucidated in randomized controlled trials. In an early study [177], the effects of long-term supplementation with trace elements (zinc 20 mg, selenium 100 μg) with or without antioxidant vitamins (beta carotene 6 mg, vitamin C 120 mg, and vitamin E (alpha-tocopherol) 15 mg) were assessed in institutionalized healthy older people. Supplementation with trace elements, with or without the antioxidant vitamins, increased antibody titers after the influenza vaccine. A trend for a reduced risk of respiratory tract infections was also observed, but only for supplementation with trace elements. An interventional pilot study that is currently underway is evaluating a daily MMN supplement (containing high-dose vitamin C (1000 mg), as well as vitamins D (10 μg), E (45 mg), A (700 μg), B6 (6.5 mg), B12 (9.6 μg), folate (400 μg), copper (0.9 mg), iron (5 mg), selenium (110 μg), and zinc (10 mg)) in older adults with no deficiencies in vitamins C and D or zinc [227]. In addition to its effects on micronutrient status, immune parameters (e.g., phagocytic activity, ROS generation by neutrophils, and levels of inflammatory cytokines), and quality of life, it is also assessing the effects on self-reported length and severity of illness. Results to date indicate that after 12 weeks of supplementation, the MMN supplement is well tolerated, significantly increases levels of vitamin C and zinc and the production of ROS by neutrophils. Importantly, it significantly decreases the length and severity of self-reported illness. Such studies are important to contribute to and improve the current knowledge base.

The same MMN was used in a study investigating the effects of supplementation for three months on the frequency of sick building syndrome (SBS), acute respiratory tract infections (ARTI), and diarrhea in 350 workers exposed to poor air quality in Jakarta, Indonesia [3]. The presence of any symptoms of SBS (e.g., headache, watery eyes, nasal congestion, throat irritation, dry cough, dry and itchy skin, dizziness, sickness, fatigue, inability to concentrate, and sensitivity to smell), ARTI (cough, cold, or flu), and diarrhea (soft and watery stool >3 times/day) were recorded. No placebo was used, as the control subjects (n = 138) were unaware that there was an intervention group. Results demonstrate that there was a significantly lower risk of developing many symptoms associated with SBS after MMN supplementation compared with no intervention, including headache (48.9% lower in the supplementation group compared with no intervention), sore eyes (45.5%), nasal congestion (51.9%), throat inflammation (27.2%), and tiredness/pain (40.8%) (*p* ≤ 0.005 vs. no intervention). Symptoms of ARTI (cough 46.2%, cold 39.6%, flu 36.2%) and diarrhea (64.6%) were also significantly lower after three months of supplementation (*p* ≤ 0.001).

## 6. Summary

Every stage of the immune response depends on the presence of certain micronutrients, which have synergistic roles based on their complementary modes of action. First, selected micronutrients (e.g., vitamins A, D, C, E, and zinc) are required to ensure the structural and functional integrity of external and internal surfaces of the body (i.e., the skin and all mucus membranes), which form physical and chemical barriers that represent a first line of defense against invading pathogens. Cell-mediated processes of innate immunity, such as cell proliferation, differentiation, function, movement, and the ability to mount an effective oxidative burst, rely on adequate amounts of vitamins A, D, C, E, B6, and B12, folate, iron, zinc, copper, selenium, and magnesium. Similarly, chemical responses such as activation of the complement system and the release of proinflammatory cytokines requires certain vitamins and minerals (in particular, vitamins A, D, and C, zinc, iron, and selenium). The inflammatory response bridges the gap between innate and adaptive immunity, and is regulated by vitamins A, C, E, and B6, as well as iron, zinc, and copper. Adaptive immune responses encompassing cell-mediated and humoral immunity depend again on the presence of a variety of micronutrients at all stages (i.e., lymphocyte proliferation, differentiation, and function, and humoral- and cell-mediated immune processes). At the same time, micronutrients are involved in self-protection of immune cells (via antioxidant mechanisms, e.g., vitamins C and E, zinc, iron, magnesium, copper, and selenium), inhibitory actions (vitamins D, B6, and E), and elimination of spent cells via apoptosis and clearance (limiting tissue damage, e.g., vitamin C).

Clearly, micronutrients are an integral part of the immune system, and the body needs optimal levels for effective immune function. It is well established that overt micronutrient deficiencies can adversely affect the immune system and predispose individuals to infections. It is likely that marginal deficiencies are also associated with increased risk of infections, although the effect may be less pronounced than those observed with overt deficiencies. The dietary intake of various micronutrients is inadequate worldwide, including industrialized countries, which can increase the risk of infection. In addition, mounting evidence suggests that increased intake of some micronutrients above the RDA may help optimize or maximize immune function and thus improve resistance to infection. Thus, a gap exists between dietary intakes and levels for optimal immune function, providing a rationale to supplement the diet with micronutrients to help support the immune system and reduce the risk of infection.

Current studies suggest beneficial effects regarding risk reduction for vitamin A (diarrhea and measles in children), vitamin D (RTI in adults and children), vitamin C (pneumonia in adults and children and common cold in active people), zinc (otitis media, RTI, pneumonia, and diarrhea in children), iron (RTI in children), and MMN supplementation (helminth infection in children and infection/reinfection in older people). In treating the symptoms of infection, benefits have been reported for vitamin A (non-measles pneumonia, as well as measles-associated diarrhea and RTI in children), vitamin D (tuberculosis, influenza and upper RTI in adults and children), vitamin C (common cold in adults and children and pneumonia in adults and older adults), and zinc (common cold in adults and children). In particular, an MMN consisting of seven vitamins and four trace elements significantly decreased the length, severity, and number of symptoms of self-reported illness in older people, and significantly reduced the symptoms of SBS, ARTI, and diarrhea in employees exposed to the habitual levels of pollution experienced in a big city such as Jakarta. It is important that supplementation is used within recommended safety limits [143], particularly in potentially at-risk groups such as pregnant or postpartum women (for vitamin A [228,229]) or smokers (in whom high doses of vitamin E, for example, may have an adverse effect on the risk of lung cancer [230,231]).

Thus, although some contradictory data exist, the overall available body of evidence suggests that supplementing the diet with a combination of multiple, selected, immune-supportive micronutrients may help to optimize immune function and reduce the risk of infection. It is worth continuing to investigate the efficacy of MMN supplements that contain immune-supporting micronutrients at doses higher than the RDA—especially as they are relatively low cost and readily available, and have the potential to reduce the global burden of infection.

## Figures and Tables

**Table 1:** Certain micronutrients have key roles in the immune system [2,7,8,9].

Immune Function Roles	Micronutrient	Comments
**Physical and biochemical barriers**		
Maintenance of structural and functional integrity of mucosal cells in innate barriers (e.g., skin, respiratory tract)	Vitamin A	Normal differentiation of epithelial tissue; retinoic acid essential to imprint T and B cells with gut-homing specificity and array T cells and IgA+ cells into intestinal tissues [8]. important for intestinal immune response, thus supporting the gut barrier [10,11,12]; carotenoids (either provitamin A or nonprovitamin A carotenoids) have immunoregulatory actions including reducing the toxic effects of ROS and regulating membrane fluidity and gap-junctional communication [13]
	Vitamin D	Calcitriol regulates antimicrobial proteins (cathelicidin and β-defensin), responsible for modifying intestinal microbiota to a healthier composition and supporting the gut barrier [10,14], as well as protecting the lungs against infection [15]; increases tight junction protein expression, E-cadherin, and connexion 43 in the gut [16,17,18]; maintains renal epithelial barrier function [19]; enhances corneal epithelial barrier function [20]
	Vitamin C	Promotes collagen synthesis and protects cell membranes from damage caused by free radicals, thus supporting integrity of epithelial barriers [8]; enhances keratinocyte differentiation and lipid synthesis as well as fibroblast proliferation and migration [21]
	Vitamin E	Protects cell membranes from damage caused by free radicals and support the integrity of epithelial barriers [7,8]
	Vitamins B6, B12 and folate	All involved in intestinal immune regulation (e.g., by mediating lymphocyte migration into the intestine in the case of vitamin B6, while folate is essential for the survival of regulatory T cells in the small intestine, and human gut microbes use vitamin B12 as a cofactor for metabolic pathways), thus supporting the gut barrier [10,22]
	Iron	Essential for differentiation and growth of epithelial tissue [3]
	Zinc	Helps maintain integrity of skin and mucosal membrane (e.g., cofactor for metalloenzymes required for cell membrane repair [23])
**Immune cells**		
Differentiation, proliferation, functioning, and movement of innate immune cells	Vitamin A	For example, regulates number and function of NK cells [2,8], contributes to phagocytic and oxidative burst activity of macrophages [8]
	Vitamin D	Vitamin D receptor found in, e.g., monocytes, macrophages, and DCs [7]; increases differentiation of monocytes to macrophages [3]; calcitriol promotes movement and phagocytic ability of macrophages [8,24,25,26]
	Vitamin C	Involved in proliferation, function, and movement of neutrophils, monocytes, phagocytes [2]; maintains or enhances NK cell activities and chemotaxis [2,3,8,27]; enhances phagocytosis and ROS generation, enhances microbial killing [21]; involved in apoptosis and clearance of spent neutrophils from sites of infection by macrophages [21]; attenuates extracellular trap (NET) formation, thus reducing associated tissue damage [28]
	Vitamin E	Maintains or enhances NK cell cytotoxic activity [2,3,8,27]; inhibits PGE2 production by macrophages (thus indirectly protecting T-cell function) [27,29]
	Vitamin B6	Maintains or enhances NK cell cytotoxic activity [2,8,27]
	Vitamin B12	May act as immunomodulator for cellular immunity, effects on cytotoxic cells (e.g., NK cells, cytotoxic T cells) [3]
	Folate	Maintains or enhances NK cell cytotoxic activity [2,8,27]
	Zinc	Maintains or enhances NK cell cytotoxic activity [2,8,27]; central role in cellular growth and differentiation of immune cells that have a rapid differentiation and turnover [30]; enhances the phagocytic activity of peritoneal macrophages for *E. coli* and *S. aureus* [31]; improves phagocytic capacity of monocytes [27,32]
	Iron	Forms highly-toxic hydroxyl radicals, thus involved in killing of bacteria by neutrophils; component of enzymes critical for functioning of immune cells (e.g., ribonucleotide reductase involved in DNA synthesis); involved in regulation of cytokine production and action [3]; iron-rich status promotes M2-like macrophage phenotype and negatively regulates M1 pro-inflammatory response [33]
	Copper	Role in functions of macrophages (e.g., copper accumulates in phagolysosomes of macrophages to combat certain infectious agents [34]), neutrophils and monocytes [35]; enhances NK cell activity [36]
	Selenium	Selenoproteins important for antioxidant host defense system, affecting leukocyte and NK cell function [35]
	Magnesium	Cofactor of enzymes of nucleic acid metabolism and stabilizes structure of nucleic acids; involved in DNA replication and repair [37]; roles in antigen binding to macrophages [38]; regulates leukocyte activation [38]; involved in the regulation of apoptosis [37]
Antimicrobial effects	Vitamin A	Downregulates IFNγ production [3,21]
	Vitamin D	Calcitriol regulates antimicrobial protein expression (cathelicidin and defensin), which directly kill pathogens, especially bacteria [7,39,40,41]; inhibits IFNγ production [27,42,43,44,45]
	Vitamin C	High levels can improve antimicrobial effects; increases serum levels of complement proteins [7]; role in IFNγ production [3,21]
	Zinc	Involved in complement activity; role in IFNγ production [3,21]
	Iron	Role in IFNγ production [3,21]
	Copper	Intrinsic antimicrobial properties [7]
	Selenium	Increases IFNγ production [3,21]
Roles in inflammation, antioxidant effects, and effects in oxidative burst	Vitamin A	Helps to regulate the production of IL-2 and the proinflammatory TNF-α, which activates the microbial action of macrophages; involved in phagocytic and oxidative burst activity of macrophages activated during inflammation [8]
	Vitamin D	Calcitriol increases the oxidative burst potential of macrophages [24,25,26]; increases superoxide synthesis [8]; reduces the expression of pro-inflammatory cytokines and increases the expression of anti-inflammatory cytokines by macrophages [24,46,47,48]
	Vitamin C	Maintains redox homeostasis within cells and protects against ROS and RNS during oxidative burst [8]; regenerates other important antioxidants, such as glutathione and vitamin E, to their active state [49]; modulates cytokine production and decreases histamine levels [21]
	Vitamin E	Important fat-soluble antioxidant that hinders the chain reaction induced by free radicals (chain-breaking effect) and protects cells against them [3,7]; enhances IL-2 production [3]; decreases production of PGE2 (indirectly protecting T-cell function) [50]
	Vitamin B6	Required in endogenous synthesis and metabolism of amino acids, the building blocks of cytokines [7]; helps to regulate inflammation (higher levels of the active form result in lower rates of inflammation) [35,51,52]
	Zinc	Anti-inflammatory agent [53]; helps to modulate cytokine release [3,49] by dampening the development pro-inflammatory Th17 and Th9 cells [27,54,55] and influencing the generation of cytokines such as IL-2, IL-6, and TNF-α [56,57]; has antioxidant effects that protect against ROS and reactive nitrogen species [49]; influences activity of antioxidant proteins [8]
	Iron	Involved in regulation of cytokine production and action [3]; required for generation of pathogen-killing ROS by neutrophils during oxidative burst [7]
	Copper	Accumulates at sites of inflammation [7,35]; part of copper/zinc-superoxide dismutase, a key enzyme in defense against ROS [8]; free-radical scavenger [58]; changes in copper homeostasis a crucial component of respiratory burst [8]; important for IL-2 production and response [7,35]; maintains intracellular antioxidant balance, suggesting important role in inflammatory response [8]
	Selenium	Essential for function of selenoproteins that act as redox regulators and cellular antioxidants, potentially counteracting ROS produced during oxidative stress [2]
	Magnesium	Can help to protect DNA against oxidative damage [37]; high concentrations reduce superoxide anion production [59]
Differentiation, proliferation and normal functioning of T cells	Vitamin A	Involved in development and differentiation of Th1 and Th2 cells [60]; enhances TGF-β-dependent conversion of naïve T cells into regulatory T cells [8]; plays a role in acquisition of mucosal-homing properties by T and B cells [8]
	Vitamin D	Homing of T cells to the skin [61]; calcitriol inhibits T-cell proliferation [7]; inhibitory effects mainly in adaptive immunity (e.g., Th1-cell activity) [7]; stimulatory effects in innate immunity [7]; inhibits the effector functions of T helper cells and cytotoxic T cells [27,62], but promotes the production of Tregs [27,62,63]; inhibitory effect on the differentiation and maturation of the antigen-presenting DCs, and helps program DCs for tolerance [27,64,65,66]
	Vitamin C	Roles in production, differentiation, and proliferation of T cells, particularly cytotoxic T cells [3,21]
	Vitamin E	Enhances lymphocyte proliferation and T-cell-mediated functions [3]; optimizes and enhances Th1 response [3]
	Vitamin B6	Involved in lymphocyte proliferation, differentiation, maturation, and activity [7]; maintains Th1 immune response [3]
	Vitamin B12	Involved in one-carbon metabolism (interactions with folate) [35]; facilitates production of T cells [35], such as cytotoxic T cells [3,8]; helps to regulate ratio between T helper cells and cytotoxic T cells [8]
	Folate	Supports Th 1-mediated immune response [35]
	Zinc	Induces proliferation of cytotoxic T cells [67]; involved in Th1 cytokine production and thus supports Th1 response [3]; essential for intracellular binding of tyrosine kinase to T cell receptors, required for T cell development, differentiation, and activation [49]; induces development of Treg cells and is thus important in maintaining immune tolerance [27,54,55]
	Iron	Important in differentiation and proliferation of T cells [7]; helps to regulate ratio between T helper cells and cytotoxic T cells [3]
	Copper	Roles in differentiation and proliferation of T cells [35]
	Selenium	Roles in differentiation and proliferation of T cells [35,58]; helps to improve Th cell counts [68]
**Antibodies**		
Antibody production and development	Vitamin A	Development and differentiation of Th 1 and Th2 cells [8]; maintains normal antibody-mediated Th2 response by suppressing IL-12, TNF-α, and IFN-γ production of Th1 cells [7]
	Vitamin D	Calcitriol suppresses antibody production by B cells [7]
	Vitamin C	Promotes proliferation of lymphocytes, resulting in increased generation of antibodies [21]
	Vitamin E	Suppresses Th2 response [3]
	Vitamin B6	Required in endogenous synthesis and metabolism of amino acids, the building blocks of antibodies [7]; inhibits Th2 cytokine-mediated activity [8]
	Vitamin B12	Important for antibody production and metabolism, via folate mechanism [7,8,35]; required for optimal clonal expansion [8]
	Folate	Important for antibody production and metabolism [7,8,35]
	Zinc	Involved in antibody production, particularly IgG [69,70]
	Selenium	Helps to maintain antibody levels [35]
	Magnesium	Cofactor in antibody synthesis, role in antibody-dependent cytolysis and IgM lymphocyte binding [38]
Responses to antigen	Vitamin A	Normal functioning of B cells, necessary for generation of antibody responses to antigen [7]; required for B cell-mediated IgA antibody responses to bacterial polysaccharide antigens [8]
	Vitamin D	Promotes antigen processing [8]; role in the down-regulation of MHC-II [35]
	Vitamin E	Helps to form effective immune synapses between and Th cells [27]; increases proportion of antigen-experienced memory T cells [71]
	Folate	Important for sufficient antibody response to antigens [35]
	Zinc	Involved in antibody response [8]; important in maintaining immune tolerance (i.e., the ability to recognize “self” from “non-self”) [27]
	Magnesium	Key role in antigen binding to macrophage RNA [38]; involved in antibody-dependent cytolysis [38]

Calcitriol = 1,25-dihydroxyvitamin D_3_, the active form of vitamin D. Selenoproteins are selenium-dependent enzymes. APC, antigen-presenting cell; DC, dendritic cells; IFN, interferon; IL, interleukin; MHC, major histocompatibility complex; NK, natural killer; PGE2, prostaglandin E2; RNS, reaction nitrogen species; ROS, reactive oxygen species; Th, helper T cell; TGF, transforming growth factor; TNF, tumor-necrosis factor; Tregs, regulatory T cells.

**Table 2:** Impact of micronutrient deficiencies and supplementation on immune functions.

Micronutrient	Impact of Deficiency		Impact of Supplementation on Immune Functions
	Immune Functions	Decreased Resistance to Infection(s)	
Vitamin C	Increased oxidative damage [111] Decreased DTH response [109] Impaired wound healing [21]	✓	High doses stimulate phagocytic and T-lymphocytic activity [8] Antioxidant properties protect leukocytes and lymphocytes from oxidative stress [7] Enhanced neutrophil chemotaxis, but no apparent effects on antibody production [115] In high doses, can help severely ill patients in intensive care recover more quickly [116]
		[8,21,109]	
		Increased incidence and severity of pneumonia and other infections [110,111]	
Vitamin D	Altered gut microbiota composition [14] Reduced number of lymphocytes [109,117] Reduced lymphoid organ weight [109] Impaired immune capabilities of macrophages (including antimicrobial functions) [118]	✓	Calcitriol helps to restore the immune function of macrophages [118] No significant effect on biomarkers of systemic inflammation (i.e., TNF-α, IL-6) [120]
		Especially RTI [110,118,119] Increased severity, morbidity and mortality [109] Increased risk of autoimmune diseases (e.g., type 1 diabetes, multiple sclerosis, systemic lupus erythematosus, rheumatoid arthritis) [7,8]	
Vitamin A	Altered integrity of mucosal epithelium [106,121] Impaired T and B cell movements in the intestine [122] Retinoic acid deficiency impairs microbiota composition and immune system function [11,12] Impaired innate immunity [8,106] Affects neutrophil and eosinophil functions [7,24,106] Reduced number and killing activity of NK cells [7,106] Impaired ability of macrophages to phagocytose pathogens [7] Diminished oxidative burst activity of macrophages [8] Increased production of IL-12 (promoting T-cell growth) and TNF-α (activating microbicidal action of macrophages) [8] Induces inflammation and potentiates existing inflammatory conditions [8] Decreased number and distribution of T cells [7] Altered Th1/Th2 balance, decreasing Th2 response [106] Adverse effect on growth and differentiation of B cells [7] Impaired antibody-mediated immunity [8]	✓	Retinoic acid modulates specific microbiota in the gut [75] Helps reverse adverse effects on immune functions of neutrophils, eosinophils, NK cells, and macrophages [8,9] Improves antibody titer response to vaccines [8]
		[106,122]	
		For example, diarrhea, RTI, measles, malaria) [7,110] Increased susceptibility to pathogens in mucosal epithelium (e.g., eye, respiratory and GI tracts) [8]	
Vitamin E	Impaired humoral and cell-mediated aspects of adaptive immunity, including B and T cell function [7] Reduces T cell maturation [35]	✓	Improves overall immune function [8,124] In the elderly, enhanced DTH responses and increased antibody titers [8]
		[35,123]	
Vitamin B6	Decreased IL-2 production [8] Reduced lymphoid tissue weight [109] Lymphocytopenia [8,109] General deficiencies in cell-mediated immunity [109], such as suppression of Th1 and promotion of Th2 cytokine-mediated activity [8,125] Impaired lymphocyte maturation and growth, even with marginal deficiency [24] Lowered antibody responses [24,109] Reduced responses to mitogens [109]	✓	Helps to restore cell-mediated immunity [8] Can improve lymphocyte maturation and growth, and increase numbers of T-lymphocytes [24] Large doses can improve immune response of critically ill patients [126]
		Reduced ability to respond to pathogenic challenge [35]	
Vitamin B12 *	Suppressed NK cell activity [8,22,103] Impaired DTH response [109] Significant reduction in cells with a role in cell-mediated immunity [24] Changes proportions of cytotoxic T cells and T helper cells, leading to abnormally high T helper/cytotoxic T cell ratio [8,22,103] Depressed T-cell proliferation [109] Decreased number of lymphocytes [8] Impaired antibody response [9]	✓	Increases numbers of cells with a role in cell-mediated immunity [24]
		(potentially) [127]	
Folate *	Impaired NK cytotoxicity [128] Impaired DTH response [109] Depressed T-cell proliferation [109] Inhibits proliferation of cytotoxic T cells [22] Impaired thymidine and purine synthesis (affecting DNA and RNA synthesis) and impaired immunoglobulin secretion [129] Decreased antibody response [35]	✓	Can increase innate immunity in older people [130] Alters age-associated decrease in NK-cell activity [128] Supports Th1 response [128]
		[8]	
Zinc	Impaired DTH skin responses [8,131,132] Impaired survival, proliferation and maturation of monocytes, NK cells, T and B cells [133] Impaired NK cell activity [8] Impaired phagocytosis by macrophages and neutrophils [8] Altered cytokine production, contributing to greater oxidative stress and inflammation [7,131,132] Impaired generation of oxidative burst [8] Impaired complement activity [8] Increased thymic atrophy [7,8,134] Decreased lymphocyte proliferation and function, particularly T cells [7,8,133] Alters the expression of genes related to proliferation, survival, and response of T-cells even with moderate deficiency [105] Decreased production of Th1 cytokines (IL-2, IFN-γ) [8] Imbalance in Th1/Th2 ratio [8,133] Impaired antibody response to T cell-dependent antigens [8]	✓	Beneficial effects in intestinal immune functions [10] Increases cytotoxicity of NK cells [7] Restores thymulin activity [7] Increases numbers of cytotoxic T cells [7] Reduces numbers of activated T helper cells (which can contribute to autoimmunity) [7]
		[122,135]	
		Increased risk of inflammatory disease, impaired wound healing [131,132] Increased bacterial, viral and fungal infections (particularly diarrhea and pneumonia) [110,134] Increased diarrheal and respiratory morbidity [109] Susceptibility particularly increased in older people and children [8]	
Iron	Decreased DTH response [109] Decreased NK cell activity [109] Impaired intracellular microbial killing by polymorphonuclear leukocytes [8] Lower IL-6 levels [109] Impaired cellular immunity (e.g., decreased T helper cells, increased cytotoxic T cells) [8] Decreased lymphocyte bactericidal activity [109] Decreased response to mitogens [109]	✓	Improves intracellular microbial killing and cellular immunity [8]
		[136]	
		For example, RTI more frequent and last longer in children [122] Possible protective effect in malaria in children [136] Helps reduce the incidence of diarrhea in children, in combination with vitamin A [137]	
Copper	Abnormally low neutrophil levels and reduced phagocytic ability [7,138] Reduced IL-2 and decreased T-cell proliferation even in marginal deficiency [7,35,138] Ineffective immune response to infections [8] Increased viral virulence [8]	✓	Increased ability of neutrophils to engulf pathogens [7,139] Too much copper can also negatively impact the immune response [8]
		(potentially) [7]	
Selenium	Suppression of immune function [109] Diminished NK-cell cytotoxicity [8] Impaired humoral and cell-mediated immunity [7] Decreased immunoglobulin titers [8] Impaired cell-mediated immunity [8] Increased viral virulence [7,8,110] Decreased response to vaccination [8]	✓	Improves cell-mediated immunity [8] Improves T helper cell counts [68] Enhances immune response to viruses in deficient individuals [7,8]
		Increased risk of RTI in the first 6 weeks of life in children [110]	
Magnesium	Decreased numbers of monocytes [140] Decreased NK-cell activity [140] Increased oxidative stress after strenuous exercise [140] Increased levels of cytokines such as IL-6 [38] Increased inflammation [38] Decreased T-cell ratios [140]	✓	Reduces oxidative damage to the DNA of peripheral blood lymphocytes in athletes and sedentary young men [37] Reduces leukocyte activation [59] After exercises, increases granulocyte count and post-exercise lymphopenia [142]
		For example, recurrent bacterial infection, fungal infections [141]	

* Immune system effects of vitamin B12 deficiency and folate deficiency are clinically indistinguishable [109]. DTH, delayed-type hypersensitivity; GI, gastrointestinal tract; IFN, interferon; IL, interleukin; NK, natural killer; RTI, respiratory tract infections.

**Table 3:** Life-stage-specific micronutrient deficiencies in Europe.

Select Micronutrients	Recommended Dietary Allowance [143]			Reported Mean Micronutrient Intakes, Min–Max [114]		
	**Children, M/F *^a^*** 4–8 years 9–13 years 14–18 years	**Adults, M/F** 19–50 years *^b^*	**Older age, M/F** 51 to >70 years	**Children, M/F**	**Adults, M/F** 19–50 years	**Older age, M/F** 51 to >70 years
				4–6 years		
				7–9 years		
				10–14 years		
				15–18 years		
Vitamin C, mg/day	25 45 75/65	90/75	90/75	60–157/61–157	**64**–153/**62**–153	**59**–142/**60**–160
				63–172/57–172		
				73–197/77–222		
				**71**–201/67–205		
Vitamin D, μg/day	15	15	15–20	**1.8–5.8/1.5–6.5**	**1.6–10.9/1.2–10.1**	**0.7**–15.0/**0.7**–**12.9**
				**1.5–6.4/1.5–5.1**		
				**1.5–4.8/1.2–4.5**		
				**1.8–7.5/1.5–7.1**		
Vitamin A, μg/day	400 600 900/700	900/700	900/700	400–1100/400–1200	**500**–2200/**500**–2200	**500**–2500/**400**–2300
				400–1300/400–1100		
				**400**–2400/**300**–2300		
				**400**–1800/**300**–1600		
Vitamin E, mg/day	7 11 15	15	15	**5.3**–9.8/**5.1**–9.8	**3.3**–17.7/**4.2**–16.1	**6.3**–**13.7**/**6.7**–**13.7**
				**6.3**–11.2/**5.9**–13.3		
				**5.9**–14.5/**5.6**–18.1		
				**6.8**–20.8/**6.0**–15.5		
Vitamin B6, mg/day	0.6 1.0 1.3/1.2	1.3	1.7/1.5	1.3–1.8/1.0–1.9	1.6–3.5/1.3–2.1	**1.2**–3.0/**1.2**–2.9
				1.2–2.5/1.1–1.9		
				1.2–2.8/1.1–2.7		
				1.5–3.1/1.2–2.5		
Vitamin B12, μg/day	1.2 1.8 2.4	2.4	2.4	2.7–5.3/2.6–5.0	**1.9**–9.3/**1.0**–8.8	3.1–8.2/2.5–7.5
				3.6–5.5/2.2–5.3		
				3.2–11.8/2.2–11.1		
				4.9–7.5/3.5–5.2		
Folate, μg/day	200 300 400	300–400	400	**120**–256/**109–199**	**203–494/131–392**	**139**–**343**/**121**–**335**
				**144**–290/**133**–264		
				**149**–428/**140**–360		
				**190–365/154–298**		
Zinc, mg/day	5 8 11/9	11/8	11/8	6.0–9.2/5.3–8.9	**8.6**–14.6/**6.7**–10.7	**7.5**–12.3/**6.7**–11.2
				7.0–10.9/6.4–9.4		
				**7.0**–14.6/**6.1**–13.9		
				**9.3**–15.2/**6.4**–11.0		
Iron, mg/day	10 8 11/15	8/18	8	**7.3**–10.6/**6.8**–10.6	10.6–26.9/**8.2**–22.2	10.2–25.2/8.5–20.9
				**8.4**–11.8/**7.7**–11.8		
				9.2–19.4/**7.7**–14.8		
				**10.2**–19.0/**7.8–14.0**		
Copper, μg/day	440 700 890	900	900	700–2200/700–2000	1100–2300/1000–2200	1100–1900/900–1900
				900–2800/800–2600		
				800–2900/700–2800		
				1200–3400/800–2100		
Selenium, μg/day	30 40 55	55	55	**23**–61/**24**–61	**36**–73/**31**–**54**	**39**–62/**34**–55
				**27**–41/**26**–58		
				**29**–110/**28**–104		
				**39**–59/**30–38**		
Magnesium, mg/day	130 240 410/360	400–420/310–320	420/320	171–267/166–267	**256**–465/**192**–372	**221**–**403**/**179**–348
				204–303/166–301		
				**200**–503/**181**–429		
				**260**–467/**186**–369		

*^a^* Although adequate intake values are provided by the Institute of Medicine for infants (0–12 months) and recommended dietary allowances for children (1–3 years) [143], there are scarce data regarding micronutrient deficiencies in this age groups in industrialized countries and these ages have therefore not been included in this table; *^b^* values differ in pregnancy and lactation. F, females; M, males. Reported micronutrient intakes in bold are below the recommended dietary allowances.

## Supplementary Materials

The following are available online at https://www.mdpi.com/2072-6643/12/1/236/s1, Table S1: Influence of micronutrients on the prevention and/or treatment of infections in adults and children: results from systematic reviews and meta-analyses.
