# Supplementary material for: A Review of Micronutrients and the Immune System–Working in Harmony to Reduce the Risk of Infection
Source: Nutrients. 2020 Jan 16;12(1):236. doi: 10.3390/nu12010236 (PMC7019735; doi:10.3390/nu12010236)
Supplement: Supplementary file 1 [file nutrients-12-00236-s001.pdf]

Table S1. Influence of micronutrients on the prevention and/or treatment of infections in adults and children: results from systematic reviews and meta-analyses.

Study [reference no.]	Micronutrient(s) (dosage)	Type of acute infection	Study population<br>Deficient: Y/N/both<br>No. studies in analysis	Key results obtained with supplementation	Adverse events reported	Positive for supplementation: Y/N<br>Quality of evidence
**PREVENTION**						
**Imdad 2017 [1]**	**Vitamin A**<br>(mostly 50,000–200,000 IU every 4-6 months)	Infections	Children<br>Both (mostly low- to middle-income countries)<br>47 RCTs or cluster-RCTs	• Reduced incidence of diarrhea (risk ratio 0.85; n=15 RCTs, low quality) and measles (0.50; n=6, moderate quality)<br>• No significant effect on incidence of respiratory disease or hospitalizations due to diarrhea or pneumonia<br>• 12% overall reduction in mortality due to diarrhea (risk ratio 0.88; n=9 RCTs, high quality), but no significant effect on mortality due to measles, respiratory disease, and meningitis	• Increased risk of vomiting within first 48 h of taking large doses (rate ratio 1.97; n=4 RCTs, moderate-quality)	**Y**<br>(diarrhea and measles)<br>++
**Mathew 2010 [2]**	**Vitamin A**<br>(5–20 mg/day)	Pneumonia	Children<br>Both (mostly low- to middle-income countries)<br>11 RCTs	• No difference between supplementation and placebo for no. children with community-acquired pneumonia, incidence per person-time, or pneumonia mortality	Possible harm to those with adequate serum retinol baseline levels	**N**<br>+++
**Chen 2008 [3]**	**Vitamin A**<br>(varied)	Lower RTI	Children<br>Both<br>10 RCTs	• No significant effect on incidence or prevalence of symptoms (n=8 studies)<br>• No differences and no protective effect (n=3)<br>• Significant reduction in incidence in children with poor nutritional status or weight, but increased incidence in healthy children (n=2)	• Increased incidence of acute lower RTI (n=1)<br>• Increase in cough and fever (n=1), and symptoms of cough and rapid breathing (n=2)<br>• Fewer side effects and equal benefit with low vs. high dose	**N**<br>(unless deficient)<br>++
**Autier 2017 [4]**	**Vitamin D**<br>(10-20 μg/day)	Upper RTI	Adults & children<br>Both<br>25 MAs	• Supplementation might help prevent common upper RTI (RR 0.88), but not pneumonia or tuberculosis)<br>• Greater effect in those with low vitamin D status	NS	**Y**<br>(especially if low status)<br>+++ (excluded suboptimal trials)

Study [reference no.]	Micronutrient(s) (dosage)	Type of acute infection	Study population<br>Deficient: Y/N/both<br>No. studies in analysis	Key results obtained with supplementation	Adverse events reported	Positive for supplementation: Y/N<br>Quality of evidence
**Martineau 2017 [5]**	**Vitamin D** (varied)	RTI	Adults & children<br>Both<br>25 RCTs	• Reduced risk of RTI (OR 0.88)<br>• Protective effects with daily or weekly doses, but not in those receiving additional boluses<br>• Greater protective effects in those with low vs. high vitamin D status (OR 0.30 vs. 0.75)	No effects on rate	**Y**<br>(especially if low status)<br>+++
**Rejnmark 2017 [6]**	**Vitamin D** (varied)	RTI	Adults & children<br>Mostly baseline >50 nmol/l<br>7 MAs & 30 RCTS	• Beneficial effect on RTI (n=3 MAs & n=9 RCTs)<br>• Wide range of ages included, from newborns to older people	NS	**Y**<br>+++
**Vuichard Gysin 2016 [7]**	**Vitamin D** (300–3700 IU/day)	RTI	Adults & children<br>Both<br>15 RCTs	• Reduction in RTI risk, but not clinically significant (RR 0.94)	Mild hypercalcemia in studies with concomitant calcium administration	**N**<br>+
**Yakoob 2016 [8]**	**Vitamin D** (400 IU/day or 100,000 IU quarterly)	Infections	Children<br>Both<br>4 RCTs	• No significant difference in occurrence of pneumonia (rate ratio 1.06) or diarrhea vs. non-supplemented children	None reported	**N**<br>++
**Xiao 2015 [9]**	**Vitamin D** (varied)	RTI	Children<br>NS<br>7 RCTs	• No significant reduction in RTI risk (RR 0.79), including pneumonia (RR 1.06), or rate of hospital admissions due to RTI	NS	**N**<br>++
**Bergman 2013 [10]**	**Vitamin D** (300–3653 IU/day)	RTI	Adults & children<br>Both<br>11 PCTs	• Protective effect against RTI (OR 0.64)<br>• Protective effect significantly larger in studies using once-daily dosing (OR 0.51) vs. bolus doses (OR 0.86)	None considered related to study drug	**Y**<br>+++
**Charan 2012 [11]**	**Vitamin D** (400–2000 IU/day)	RTI	Adults & children<br>NS<br>5 RCTs	• RTI events significantly lower (OR 0.58), with benefits in adults (OR 0.65) and children (OR 0.58)	NS	**Y**<br>+++
**Yamshchikov 2009 [12]**	**Vitamin D** (varied)	Infections	Adults & children<br>Both<br>10 RCTs, 3 non-RCTs	• Potential benefits in tuberculosis, influenza, upper RTI<br>• No clear benefits in bacterial infections	Serious adverse events such as hypercalcemia were rare (n=3 patients)	**??**<br>++

Study [reference no.]	Micronutrient(s) (dosage)	Type of acute infection	Study population<br>Deficient: Y/N/both<br>No. studies in analysis	Key results obtained with supplementation	Adverse events reported	Positive for supplementation: Y/N<br>Quality of evidence
**Ghouri 2018 [13]**	**Vitamin C**<br>(100 mg/day)	UTI	Pregnancy<br>NS<br>1 RCT	• Significantly fewer UTI infections in supplemented (12.7%) vs. non-supplemented women (29.1%)	No harmful effects reported	**Y**<br>(limited evidence)<br>++
**Hemilä 2013 [14]**	**Vitamin C**<br>(≥0.2 g/day)	Common cold	Adults & children<br>NS<br>29 PCTs in general population, 5 PCTs after severe physical exercise	• Pooled RR of developing a cold was 0.97 (i.e., no benefit)<br>• Pooled RR in marathon runners, skiers and soldiers was 0.48 (i.e., half the risk vs. non-supplemented participants)	Similar rates of (non-serious) events as placebo	**N**<br>(general population)<br>**Y**<br>(after severe physical exercise)<br>+++
**Hemilä 2013 [15]**	**Vitamin C**<br>(NS)	Pneumonia	Adults & children<br>Both<br>3 controlled trials	• Significant reduction in pneumonia risk (≥80%)	None noteworthy	**Y**<br>(especially if dietary intake is low; limited evidence)<br>++
**Moreira 2007 [16]**	**Vitamin C (± vitamin E or zinc)**<br>(vitamin C 0.3-2.0 g/day)	Upper RTI	Adults (athletes)<br>NS<br>13 RCTs and PCTs	• Pooled rate ratio vs. placebo for upper RTI 0.49 (n=2 trials)<br>• No additional benefits with other antioxidants<br>• Augmented lymphocyte increase after exercise, attenuated serum cortisol and increases in inflammatory cytokines with ≥1 g/day	Not assessed in the trials	**Y**<br>(limited evidence)<br>+
**Gulani 2014 [17]**	**Zinc**<br>(5-50 mg/day)	Otitis media	Children<br>Both (mostly low- or middle-income countries)<br>10 RCTs	• Significantly lower incidence rate in younger children <6 months (rate ratio 0.69; n=1 study) and fewer episodes in undernourished children (n=1)<br>• No significant difference in number of children with definite otitis media (n=2); no differences in breast-fed infants (n=1) or those with HIV (n=1); no difference in number of children experiencing >1 episode (n=1)	None, apart from vomiting on administration	**Y**<br>(younger or undernourished children)<br>+++

Study [reference no.]	Micronutrient(s) (dosage)	Type of acute infection	Study population<br>Deficient: Y/N/both<br>No. studies in analysis	Key results obtained with supplementation	Adverse events reported	Positive for supplementation: Y/N<br>Quality of evidence
**Mayo-Wilson 2014 [18,19]**	**Zinc ± iron**<br>(zinc 5 to ≥20 mg/day)	Infections	Children<br>Both (mostly low- or middle-income countries)<br>73 RCTs	• No reduction in risk of RTI or malaria<br>• Reduced risk of death from RTI (rate ratio 0.86), diarrhea (0.95), and malaria (0.90)<br>• No significant benefits with zinc + iron	• Increase in number of participants with ≥1 vomiting episode (rate ratio 1.29)<br>• Negative effect on copper status	**N**<br>(not for prevention of infection; harms may outweigh benefits)<br>++
**Roth 2010 [20]**	**Zinc**<br>(20–140 mg/week)	Lower RTI	Children<br>Both (developing countries)<br>10 RCTs	• Reduction in incidence of lower RTI defined by specific clinical criteria (IRR 0.65), but no effect on lower-specificity definitions based on caregiver report (IRR 1.01) or WHO-defined 'non-severe pneumonia' (0.96)	NS	**Y**<br>(but depends on how lower RTI is defined)<br>++
**Aggarwal 2007 [21]**	**Zinc**<br>(varied)	Diarrhea & RTI	Children<br>NS<br>15 RCTs	• Significantly fewer attacks of severe diarrhea or dysentery (rate ratio 0.85), persistent diarrhea (0.75), and lower RTI or pneumonia (0.80)<br>• Fewer episodes of diarrhea (rate ratio 0.86) and RTI (0.92)<br>• Fewer total days with diarrhea (0.86) but not with respiratory illness (0.95)	NS	**Y**<br>+++
**Gera 2002 [22]**	**Iron**<br>(varied)	Infections	Children<br>Both (mostly low- to middle-income countries)<br>28 RCTs	• No increase in the overall risk of infection (IRR 1.02)<br>• Significant protective effect against RTI (IRR 0.92; n=4 RCTs)<br>• Increase in risk of developing diarrhea (IRR 1.11)<br>• No significant effect on other illnesses and malarial parasitemia	None	**Y**<br>(for RTI; limited evidence)<br>++ and +++
**de Gier 2014 [23]**	**MMN**<br>(NS)	Helminth infections	Children<br>Both (low- to middle-income countries)<br>3 RCTs	• RCT meta-analysis indicated modest protective effect for MMN interventions on helminth infection and reinfection rates (OR 0.77)	NS	**Y**<br>++

Study [reference no.]	Micronutrient(s) (dosage)	Type of acute infection	Study population<br>Deficient: Y/N/both<br>No. studies in analysis	Key results obtained with supplementation	Adverse events reported	Positive for supplementation: Y/N<br>Quality of evidence
**Stephen & Avenell [24]**	**MMN**<br>(varied; some nutrients at doses >RNI [14 studies], others <RNI [8 studies])	Infection (not specified)	Adults<br>Both<br>20 RCTs (mostly placebo controlled)	Number of episodes of infection vs. non-supplemented adults:<br>• No significant differences in older adults (≥65 years)<br>• Significantly fewer episodes in younger adults (<65 years)<br>At least one infection vs. non-supplemented adults:<br>• No significant differences in older adults (RR 0.98) or younger adults (RR 0.81)<br>Supplementation may be more beneficial in undernourished older adults (≥65 years) if supplemented for >6 months	Reported in 8 studies<br>Rare, and not likely attributable to supplementation	**N**<br>(older adults)<br>**??**<br>(older, undernourished adults)<br>**Y**<br>(younger adults, for number of infection episodes)<br>++<br>(small studies)
**El-Kadiki 2005 [25]**	**MMN**<br>(varied)	Infections	Older adults<br>Both<br>8 RCTs	• Mean annual number of days spent with infection significantly reduced by 17.5 days (n=3 RCTs)<br>• OR for at least one infection was 1.10 (n=3)<br>• Infection rate ratio vs. placebo was 0.89 (i.e., slightly lower, but not significant; n=4)	Reporting was poor	**Y**<br>(but limited and weak evidence)<br>++
**TREATMENT**						
**Mathew 2010 [2]**	**Vitamin A**<br>(5–20 mg/day)	Pneumonia	Children<br>Both (mostly low- to middle-income countries)<br>11 RCTs	• No difference between supplementation and placebo for duration of hospitalization and illness, complications and side effects<br>• No benefits of adding zinc to antibiotic therapy (n=4 additional RCTs)	• Possible harm to those with adequate serum retinol baseline levels	**N**<br>+++

Study [reference no.]	Micronutrient(s) (dosage)	Type of acute infection	Study population<br>Deficient: Y/N/both<br>No. studies in analysis	Key results obtained with supplementation	Adverse events reported	Positive for supplementation: Y/N<br>Quality of evidence
**Wu 2005 [26]**	**Vitamin A** (varied, but low to high dose)	Pneumonia (non-measles)	Children<br>Both<br>6 RCTs	• No significant reduction in mortality associated with pneumonia vs. no treatment (pooled OR 1.29)<br>• No significant difference in duration of hospital stay (MD 0.08)<br>• Low-dose vitamin A significantly reduced recurrence rate of bronchopneumonia (OR 0.12)<br>• Moderate-dose vitamin A significantly reduced time to remission of signs in children with normal serum retinol (>200 ug/L)<br>• Lack of benefit of vitamin A may be disease-specific, with vitamin A only being effective when pneumonia is complicated with measles	• Disease severity after high-dose vitamin A significantly worse vs. placebo	**Y**<br>(bronchopneumonia recurrence and time to remission)<br><br>**N**<br>(mortality and hospital stay)<br><br>++
**Glasziou 1993 [27]**	**Vitamin A** (varied)	Infections	Children<br>Both (most community studies excluded overt deficiency)<br>12 RCTs	• Significant 39% reduction in death from diarrheal disease (community studies) and 70% reduction in death from respiratory disease in measles studies	NS	**Y**<br>++
**Das 2018 [28]**	**Vitamin D** (adjunct to antibiotics; varied doses)	Pneumonia	Children<br>Both (low-income countries)<br>7 RCTs	• Inconclusive results vs. placebo: time to resolution of acute illness (h) (MD –0.95; n=3 RCTs), mortality rate (risk ratio 0.97; n=1); duration of hospitalization (MD 0.49; n=4), time to resolution of fever (MD 1.66; n=4)	None	**??**<br>+
**Yamshchikov 2009 [12]**	**Vitamin D** (varied)	Infections	Adults & children<br>Both<br>10 RCTs, 3 non-RCTs	• Potential benefits in tuberculosis, influenza, upper RTI<br>• No clear benefits in bacterial infections	Serious adverse events such as hypercalcemia were rare (n=3 patients)	**??**<br>++

Study [reference no.]	Micronutrient(s) (dosage)	Type of acute infection	Study population<br>Deficient: Y/N/both<br>No. studies in analysis	Key results obtained with supplementation	Adverse events reported	Positive for supplementation: Y/N<br>Quality of evidence
**Ran 2018 [29]**	**Vitamin C** (4–8 g/day therapeutic dose from first day of illness)	Common cold	Adults & children<br>NS<br>9 RCTs	• Therapeutic doses at cold onset vs. placebo significantly reduced cold duration (MD –0.56), shortened time of confinement indoors (MD –0.41), and relieved cold symptoms including chest pain (MD –0.40), fever (MD –0.45), and chills (MD –0.36)	NS	**Y**<br>+++ (two +)
**Hemilä 2013 [14]**	**Vitamin C** (≥0.2 g/day)	Common cold	Adults & children<br>NS<br>31 PCTs	• Cold duration reduced by 8% in adults and 14% in children; higher doses (1–2 g/day) shortened colds by 18% in children<br>• Severity of colds also reduced<br>• No consistent benefit on duration or severity in therapeutic trials (but none were in children)	Similar rates of (non-serious) events as placebo	**Y**<br>(on an individual basis, and in children)<br>+++
**Hemilä 2013 [15]**	**Vitamin C** (NS)	Pneumonia	Adults<br>Both<br>3 PCTs	• Lower mortality and reduced severity in older people, particularly those who were the most ill (n=1 study)<br>• Dose-dependent reduction in duration of pneumonia (n=1)<br>• No therapeutic benefits in hospital-acquired pneumonia (in burns victims) (n=1)	None that were noteworthy	**Y**<br>(especially if plasma levels are low)<br>++
**Caicedo Ochoa 2018 [30]**	**Vitamin C ± vitamin E** (varied; added to antibiotic therapy)	*Helicobacter pylori*	Adults (but data not available in 3 studies)<br>NS<br>10 RCTs	• No relationship with eradication of therapy with vitamins C + E (OR 1.98) or with vitamin C alone (OR 1.17)	Nausea most commonly reported	**N**<br>(but high risk of bias and different dosages used)<br>+
**Li 2011 [31]**	**Vitamin C ± vitamin E**	*Helicobacter pylori*	Adults<br>NS	• Non-significant difference in eradication rate of *H. pylori* between	Nausea, diarrhea, headache and skin rash	**N**<br>(but limited evidence)

Study [reference no.]	Micronutrient(s) (dosage)	Type of acute infection	Study population<br>Deficient: Y/N/both<br>No. studies in analysis	Key results obtained with supplementation	Adverse events reported	Positive for supplementation: Y/N<br>Quality of evidence
	(0.25–1 g/day ± 0.1–0.4 g/day; added to eradication regime)		6 RCTs	addition of vitamins C & E (risk ratio 0·93; n=3 RCTs) or vitamin C alone (0.83; n=3)		+
**Tie 2016 [32]**	**Zinc**<br>(mostly 10–20 mg/day; adjunct to antibiotics)	Pneumonia	Children<br>Both (low- to middle-income countries)<br>9 RCTs	• Failure to reduce time to recovery from severe pneumonia (HR 1·04), hospital length of stay (HR 1·04), treatment failure (RR 0·95), or change of antibiotics (RR 1·07)<br>• No significant difference vs. antibiotics alone for death rate or recovery times of severe pneumonia indicators	No significant difference in rate of adverse events	**N**<br>++
**Das 2012 [33]**	**Zinc**<br>(varied, mostly 10–20 mg/day; adjunct to antibiotics)	Pneumonia	Children (<5 years)<br>Both (developing countries)<br>7 PCTs	• No significant difference between zinc and placebo regarding time of resolution of severe illness, duration of hospitalization, duration of resolution of hypoxia, chest indrawing or tachypnoea, change of antibiotics and treatment failure rates	No significant adverse events	**N**<br>(but different methodologies may have affected results)<br>+++
**Haider 2011 [34]**	**Zinc**<br>(10-20 mg/day)	Pneumonia	Children<br>Both (developing countries)<br>4 RCTs	• No significant effect in all pneumonia on time-to-clinical recovery (HR 1.02)<br>• No significant effect in severe pneumonia on time-to-recovery from tachypnoea (respiratory rate >50 breaths per minute) (HR 1.13) or time-to-recovery from chest in-drawing (HR 1.08)<br>• In severe pneumonia, non-significant effect on time-to-hospital discharge (HR 1.04)	No serious adverse events	**N**<br>++

Study [reference no.]	Micronutrient(s) (dosage)	Type of acute infection	Study population<br>Deficient: Y/N/both<br>No. studies in analysis	Key results obtained with supplementation	Adverse events reported	Positive for supplementation: Y/N<br>Quality of evidence
**Hemilä 2011 [35]**	**Zinc**<br>(30–207 mg/day)	Common cold duration	Adults & children<br>NS<br>13 PCTs	• No effect with daily zinc dose <75 mg/day (n=5 PCTs)<br>• 42% reduction in cold duration with zinc acetate >75 mg/day (n=3)<br>• 20% reduction in cold duration with other zinc salts >75 mg/day (n=5)	Bad taste after administration	**Y**<br>+++
**Grobler 2016 [36]**	**MMN**<br>(varied, but up to 10x dietary reference intake)	Tuberculosis	Adults & children<br>Both<br>6 RCTs (MMN)	• Routine MMN may have little or no effect on mortality in HIV-negative people with tuberculosis (risk ratio 0.86; n=4 RCTs) or HIV-positive people not taking antiretroviral therapy (0.92; n=3)<br>• Insufficient evidence whether MMN improves cure (no trials), treatment completion (0.99; n=1), or proportion of people who remain sputum positive during first 8 weeks (0.92; n=2)	No significant differences between MMN and placebo	**N**<br>(but limited evidence)<br>++

+, mainly reported as low quality studies; ++, reported as a mixture of low and moderate quality studies; +++ mainly reported as high quality studies; different definitions of 'low', 'moderate', or 'high' quality were used within the analyses.

HR, hazard ratio; IRR, incidence rate ratio; MA, meta-analyses; MD, mean difference; MMN, multiple micronutrients; NS, not stated; OR, odds ratio; PCTs, placebo-controlled trials (not necessarily randomized); RCTs, randomized controlled trials; RNI, recommended nutrient intakes; RR, relative risk; RTI, respiratory tract infection; UTI, urinary tract infection; WHO, World Health Organization.

References

1. Imdad, A.; Mayo-Wilson, E.; Herzer, K.; Bhutta, Z.A. Vitamin A supplementation for preventing morbidity and mortality in children from six months to five years of age. *Cochrane Database Syst Rev* **2017**, *3*, Cd008524.
2. Mathew, J.L. Vitamin A supplementation for prophylaxis or therapy in childhood pneumonia: a systematic review of randomized controlled trials. *Indian Pediatr* **2010**, *47*, 255-261.
3. Chen, H.; Zhuo, Q.; Yuan, W.; Wang, J.; Wu, T. Vitamin A for preventing acute lower respiratory tract infections in children up to seven years of age. *Cochrane Database Syst Rev* **2008**, Cd006090.
4. Autier, P.; Mullie, P.; Macacu, A.; Dragomir, M.; Boniol, M.; Coppens, K.; Pizot, C.; Boniol, M. Effect of vitamin D supplementation on non-skeletal disorders: a systematic review of meta-analyses and randomised trials. *Lancet Diabetes Endocrinol* **2017**, *5*, 986-1004.
5. Martineau, A.R.; Jolliffe, D.A.; Hooper, R.L.; Greenberg, L.; Aloia, J.F.; Bergman, P.; Dubnov-Raz, G.; Esposito, S.; Ganmaa, D.; Ginde, A.A., *et al.* Vitamin D supplementation to prevent acute respiratory tract infections: systematic review and meta-analysis of individual participant data. *BMJ* **2017**, *356*, i6583.
6. Rejnmark, L.; Bislev, L.S.; Cashman, K.D.; Eiriksdottir, G.; Gaksch, M.; Grubler, M.; Grimnes, G.; Gudnason, V.; Lips, P.; Pilz, S., *et al.* Non-skeletal health effects of vitamin D supplementation: A systematic review on findings from meta-analyses summarizing trial data. *PLoS One* **2017**, *12*, e0180512.
7. Vuichard Gysin, D.; Dao, D.; Gysin, C.M.; Lytvyn, L.; Loeb, M. Effect of Vitamin D3 Supplementation on Respiratory Tract Infections in Healthy Individuals: A Systematic Review and Meta-Analysis of Randomized Controlled Trials. *PLoS One* **2016**, *11*, e0162996.
8. Yakoob, M.Y.; Salam, R.A.; Khan, F.R.; Bhutta, Z.A. Vitamin D supplementation for preventing infections in children under five years of age. *Cochrane Database Syst Rev* **2016**, *11*, Cd008824.
9. Xiao, L.; Xing, C.; Yang, Z.; Xu, S.; Wang, M.; Du, H.; Liu, K.; Huang, Z. Vitamin D supplementation for the prevention of childhood acute respiratory infections: a systematic review of randomised controlled trials. *Br J Nutr* **2015**, *114*, 1026-1034.
10. Bergman, P.; Lindh, Å.; Björkhem-Bergman, L.; Lindh, J. Vitamin D and respiratory tract infections: a systematic review and meta-analysis of randomized controlled trials. *PloS One* **2013**, *8*, e65835.
11. Charan, J.; Goyal, J.; Saxena, D.; Yadav, P. Vitamin D for prevention of respiratory tract infections: A systematic review and meta-analysis. *Pharmacol Pharmacother* **2012**, *3*, 300-303.
12. Yamshchikov, A.V.; Desai, N.S.; Blumberg, H.M.; Ziegler, T.R.; Tangpricha, V. Vitamin D for treatment and prevention of infectious diseases: a systematic review of randomized controlled trials. *Endocr Pract* **2009**, *15*, 438-449.
13. Ghouri, F.; Hollywood, A.; Ryan, K. A systematic review of non-antibiotic measures for the prevention of urinary tract infections in pregnancy. *BMC Pregnancy Childbirth* **2018**, *18*, 99.
14. Hemilä, H.; Chalker, E. Vitamin C for preventing and treating the common cold. *Cochrane Database Syst Rev* **2013**, *(1):CD000980*.
15. Hemila, H.; Louhiala, P. Vitamin C for preventing and treating pneumonia. *Cochrane Database Syst Rev* **2013**, Cd005532.
16. Moreira, A.; Kekkonen, R.A.; Delgado, L.; Fonseca, J.; Korpela, R.; Haahtela, T. Nutritional modulation of exercise-induced immunodepression in athletes: a systematic review and meta-analysis. *Eur J Clin Nutr* **2007**, *61*, 443-460.
17. Gulani, A.; Sachdev, H.S. Zinc supplements for preventing otitis media. *Cochrane Database Syst Rev* **2014**, Cd006639.
18. Mayo-Wilson, E.; Imdad, A.; Junior, J.; Dean, S.; Bhutta, Z.A. Preventive zinc supplementation for children, and the effect of additional iron: a systematic review and meta-analysis. *BMJ Open* **2014**, *4*, e004647.
19. Mayo-Wilson, E.; Junior, J.A.; Imdad, A.; Dean, S.; Chan, X.H.; Chan, E.S.; Jaswal, A.; Bhutta, Z.A. Zinc supplementation for preventing mortality, morbidity, and growth failure in children aged 6 months to 12 years of age. *Cochrane Database Syst Rev* **2014**, Cd009384.
20. Roth, D.E.; Richard, S.A.; Black, R.E. Zinc supplementation for the prevention of acute lower respiratory infection in children in developing countries: meta-analysis and meta-regression of randomized trials. *Int J Epidemiol* **2010**, *39*, 795-808.
21. Aggarwal, R.; Sentz, J.; Miller, M.A. Role of zinc administration in prevention of childhood diarrhea and respiratory illnesses: a meta-analysis. *Pediatrics* **2007**, *119*, 1120-1130.
22. Gera, T.; Sachdev, H.P. Effect of iron supplementation on incidence of infectious illness in children: systematic review. *BMJ* **2002**, *325*, 1142.
23. de Gier, B.; Campos Ponce, M.; van de Bor, M.; Doak, C.M.; Polman, K. Helminth infections and micronutrients in school-age children: a systematic review and meta-analysis. *Am J Clin Nutr* **2014**, *99*, 1499-1509.
24. Stephen, A.I.; Avenell, A. A systematic review of multivitamin and multimineral supplementation for infection. *J Hum Nutr Diet* **2006**, *19*, 179-190.
25. El-Kadiki, A.; Sutton, A.J. Role of multivitamins and mineral supplements in preventing infections in elderly people: systematic review and meta-analysis of randomised controlled trials. *BMJ* **2005**, *330*, 871.
26. Wu, T.; Ni, J.; Wei, J. Vitamin A for non-measles pneumonia in children. *Cochrane Database Syst Rev* **2005**, Cd003700.
27. Glasziou, P.P.; Mackerras, D.E. Vitamin A supplementation in infectious diseases: a meta-analysis. *BMJ* **1993**, *306*, 366-370.
28. Das, R.R.; Singh, M.; Naik, S.S. Vitamin D as an adjunct to antibiotics for the treatment of acute childhood pneumonia. *Cochrane Database Syst Rev* **2018**, *7*, Cd011597.
29. Ran, L.; Zhao, W.; Wang, J.; Wang, H.; Zhao, Y.; Tseng, Y.; Bu, H. Extra dose of vitamin C based on a daily supplementation shortens the common cold: A meta-analysis of 9 randomized controlled trials. *BioMed Research International* **2018**, *2018*, 12.

30. Caicedo Ochoa, E.Y.; Quintero Moreno, C.O.; Mendez Fandino, Y.R.; Sanchez Fonseca, S.C.; Cortes Motta, H.F.; Guio Guerra, S.A. Assessment of the use of vitamin C and E supplements concomitantly to antibiotic treatment against Helicobacter pylori: A systematic review and meta-analysis. *Med Clin (Barc)* **2018**, *151*, 45-52.
31. Li, G.; Li, L.; Yu, C.; Chen, L. Effect of vitamins C and E supplementation on Helicobacter pylori eradication: a meta-analysis. *Br J Nutr* **2011**, *106*, 1632-1637.
32. Tie, H.T.; Tan, Q.; Luo, M.Z.; Li, Q.; Yu, J.L.; Wu, Q.C. Zinc as an adjunct to antibiotics for the treatment of severe pneumonia in children <5 years: a meta-analysis of randomised-controlled trials. *Br J Nutr* **2016**, *115*, 807-816.
33. Das, R.R.; Singh, M.; Shafiq, N. Short-term therapeutic role of zinc in children < 5 years of age hospitalised for severe acute lower respiratory tract infection. *Paediatr Respir Rev* **2012**, *13*, 184-191.
34. Haider, B.A.; Lassi, Z.S.; Ahmed, A.; Bhutta, Z.A. Zinc supplementation as an adjunct to antibiotics in the treatment of pneumonia in children 2 to 59 months of age. *Cochrane Database Syst Rev* **2011**, Cd007368.
35. Hemilä, H. Zinc lozenges may shorten the duration of colds: a systematic review. *Open Respir Med J* **2011**, *5*, 51-58.
36. Grobler, L.; Nagpal, S.; Sudarsanam, T.D.; Sinclair, D. Nutritional supplements for people being treated for active tuberculosis. *Cochrane Database Syst Rev* **2016**, Cd006086.
